# Preparation and Properties of Deep Eutectic Solvent-Modified Soy Protein and Soy Protein–Chitosan Composite Films

**DOI:** 10.3390/polym18212743

**Published:** 2026-11-04

**Authors:** Huachun Qi, Xiaoqi Wang, Yuetong Lin, Honggang Zhao, Jiuyin Pang, Yaoxing Sun

**Affiliations:** College of Materials Science and Engineering, Beihua University, Jilin 132013, China; linda20220517@163.com (H.Q.); 15610981681@163.com (X.W.); linyuetong0128@163.com (Y.L.); mailzhaoyang163@aliyun.com (H.Z.); pangjiuyin@163.com (J.P.)

**Keywords:** chitosan, soy protein isolate, deep eutectic solvent, dual crosslinking, antioxidant film

## Abstract

Conventional non-biodegradable plastic packaging causes severe environmental pollution, driving the demand for degradable biomass food packaging. Chitosan (CS) is an ideal green film material but suffers weak mechanical, water-resistance and antioxidant properties. Soy protein isolate (SPI) is cheap and abundant; however, its compact molecular structure leads to poor miscibility with pure CS. Herein, ultrasonic-assisted deep eutectic solvent (DES) pretreatment was used to unfold SPI’s protein structure and release active sites for stronger SPI–CS interfacial interactions. Citric acid and transglutaminase (TG) served as dual green crosslinkers, while carvacrol (Car) and gallic acid (GA) were added as natural polyphenol antioxidants. Single-factor and Box–Behnken response surface experiments determined the optimal film-forming parameters: a citric acid mass ratio of 0.84, a TG dosage of 2.60 U∙g^−1^, and a drying temperature of 65 °C. The optimized DES–SPI–CS film possessed a tensile strength of 18.79 MPa and an elongation at break of 41.39%, with greatly enhanced water resistance and thermal stability. After co-loading Car and GA, the composite film reached 79.33% DPPH radical-scavenging activity. Twelve-day cherry tomato preservation tests confirmed its capacity to slow vitamin C loss and fruit decay. This work provides a sustainable integrated strategy combining DES modification, dual crosslinking and natural antioxidant doping to fabricate multifunctional bio-based packaging, offering theoretical support for biodegradable film substitution of disposable plastics.

## 1. Introduction

Conventional petroleum-based plastic packaging materials trigger serious ecological contamination, which has spurred growing research interest in renewable, biodegradable food-packaging alternatives [1,2]. Soy protein isolate (SPI), an inexpensive and readily available biomass protein, possesses tightly packed intrinsic molecular architectures that shield reactive functional groups [3,4]; simple physical blending of SPI with chitosan (CS, deacetylation degree ≥95%) yields weak interfacial compatibility, thereby compromising the comprehensive performance of resultant composite films [5,6,7,8,9,10,11,12]. Chitosan stands out as an eco-friendly film-forming matrix, yet neat chitosan films exhibit inferior mechanical robustness and unsatisfactory water resistance, restricting their practical industrial application [13,14,15,16,17]. Existing investigations have confirmed that the aggregation behavior of plant proteins dominates their emulsification and film-forming properties, and enzymatic modification serves as an efficient strategy to tune protein functional attributes [18,19]. In addition, systematic review articles have elaborated the physicochemical traits and broad application prospects of diverse deep eutectic solvent (DES) systems, laying a theoretical foundation for protein pretreatment design [20,21,22].

To improve the packaging performance of neat chitosan films, three main modification strategies have been extensively explored in the recent literature. The first is blending with other biodegradable polymers, such as poly(vinyl alcohol) (PVA), which can enhance the mechanical integrity and film-forming ability of chitosan-based matrices through intermolecular hydrogen bonding [23]. The second is the use of green solvents, including ionic liquids and deep eutectic solvents, to disrupt the strong intermolecular associations of polysaccharides and proteins, thereby improving their solubility and processability [21,22]. The third is the incorporation of plasticizers such as glycerol and sorbitol to modulate film flexibility and reduce brittleness [24]. Although these strategies individually improve certain properties of chitosan films, most of them focus on directly modifying the CS matrix or simply adding one functional component, without resolving the intrinsic interfacial incompatibility between the protein and polysaccharide phases. In this work, we address this knowledge gap by pretreating SPI with inorganic ternary DES coupled with ultrasonication to unfold its compact structure and expose reactive sites, and subsequently constructing a dual-crosslinked SPI–CS network with citric acid and transglutaminase, followed by dual-polyphenol co-loading. This integrated strategy simultaneously improves interfacial compatibility, mechanical performance, water resistance, and antioxidant activity, which is difficult to achieve through any single strategy reported previously.

Ultrasonic cavitation coupled with DES treatment can unfold the higher-order spatial structures of SPI to expose embedded active functional moieties, effectively resolving the poor miscibility issue between SPI and CS [25,26]. Citric acid and transglutaminase (TG) act as dual eco-friendly crosslinkers to construct compact biopolymer networks, while natural carvacrol (Car) and gallic acid (GA) are incorporated to endow composite films with antioxidant activity for fresh fruit preservation [27,28,29].

Beyond functioning as simple antioxidant additives, plant-derived polyphenols can serve as integral co-components within multicomponent biopolymer networks. Their abundant phenolic hydroxyl groups are capable of forming hydrogen-bonding and electrostatic interactions with both protein and polysaccharide chains, thereby modulating crosslinking density, microstructure, and physicochemical performances of composite films, rather than only contributing bioactive functions. For multicomponent SPI–CS–polyphenol systems, gallic acid and carvacrol have been reported to interact with biopolymer backbones and further tune mechanical, barrier and thermal properties, as well as providing radical-scavenging activity, which highlights their dual roles as both structural participants and functional constituents in edible packaging materials. Nevertheless, most existing investigations focus on single-polyphenol incorporation; fewer reports have explored the combined effect of two distinct polyphenols as co-components within DES-pretreated and dual-crosslinked SPI–CS composite matrices.

In this study, ultrasound-assisted DES pretreatment was utilized to modify native SPI. Single-factor experiments and Box–Behnken response surface methodology (BBD-RSM) were jointly employed to optimize the preparation parameters of dual-crosslinked SPI–CS composite films [30]. A full set of characterizations covering mechanical performance, water resistance, thermal stability and DPPH free radical-scavenging capacity was implemented, and cherry tomato storage trials were conducted to validate the practical fresh-keeping efficacy of as-prepared films. This study establishes an integrated modification pathway for multifunctional edible packaging films, providing theoretical support for the substitution of single-use plastic packaging with protein–polysaccharide biocomposites [31,32].

Most prior research on SPI–CS packaging films only adopts a single modification strategy, such as choline chloride-based binary DES treatment, individual enzymatic crosslinking, or single-antioxidant incorporation. Rare reports integrate inorganic ternary ZnCl_2_-H_3_PO_4_-H_2_O ultrasonic DES pretreatment, citric acid/TG dual green crosslinking, and dual-polyphenol (carvacrol/gallic acid) doping within one unified manufacturing workflow. Compared with the published literature, this study puts forward three innovative findings:

Against this background, this work aims to fabricate multifunctional SPI–CS composite films as candidate food-packaging materials via ultrasonic-assisted inorganic ternary DES pretreatment combined with citric acid-TG dual crosslinking. We systematically characterize the structure, mechanical performance, water-resistance and thermal stability of the resulting films. Additionally, two natural polyphenols (Car and GA) are incorporated as co-components to construct active antioxidant composite films. Finally, cherry tomato preservation experiments are performed to verify the practical fresh-keeping potential of the obtained biopolymer films. This study is expected to provide experimental basis for developing low-cost, fully-biodegradable food-packaging materials.

Compared with previously reported SPI–CS packaging films, this work advances the field through an integrated, one-step manufacturing strategy that simultaneously addresses three long-standing limitations: the poor interfacial compatibility between SPI and CS, the inadequate mechanical robustness and water resistance of neat polysaccharide films, and the lack of antioxidant functionality. Rather than adopting a single modification route, we combine inorganic ternary ZnCl_2_-H_3_PO_4_-H_2_O DES pretreatment with ultrasonication to unfold SPI and expose active sites, citric acid/TG dual green crosslinking to construct a compact biopolymer network, and dual-polyphenol co-loading to provide sustained antioxidant activity. This synergistic integration yields films with markedly improved mechanical, water-resistance, thermal and antioxidant performance that are difficult to achieve using any individual strategy. The resulting edible films are particularly promising for active packaging of fresh, perishable produce such as cherry tomatoes, and can be potentially extended to other fruits and vegetables with high respiration rates.

## 2. Materials and Methods

### 2.1. Materials and Reagents

SPI was supplied by Zhejiang Yinuo Biotechnology Co., Ltd., Hangzhou, China. Chitosan (CS, deacetylation degree ≥95%, weight-average molecular weight = 220 kDa), glacial acetic acid (99.5%), sorbitol (≥98%), citric acid, carvacrol (Car, ≥99%), gallic acid (GA, ≥99%), 1,1-diphenyl-2-picrylhydrazyl (DPPH, analytical grade), 2,6-dichlorophenolindophenol (≥97%) and L-ascorbic acid were purchased from Aladdin Industrial Corporation, Shanghai, China. Zinc chloride (ZnCl, AR), phosphoric acid (H_3_PO_4_, 85%, AR), Coomassie brilliant blue G-250, bovine serum albumin and absolute ethanol were obtained from Anhui Zesheng Technology Co., Ltd., Hefei, China. Transglutaminase (TG, food grade) was provided by Shanghai Xintai Industrial Co., Ltd., Shanghai, China. Glycerol (analytical grade) was sourced from Sinopharm Chemical Reagent Co., Ltd., Beijing, China. Fresh cherry tomatoes were purchased from a local market in Jilin, China, for fruit preservation tests.

### 2.2. Sample Preparation and Characterizations

#### 2.2.1. Preparation of DES and SPI Pretreatment

**Figure 1:**
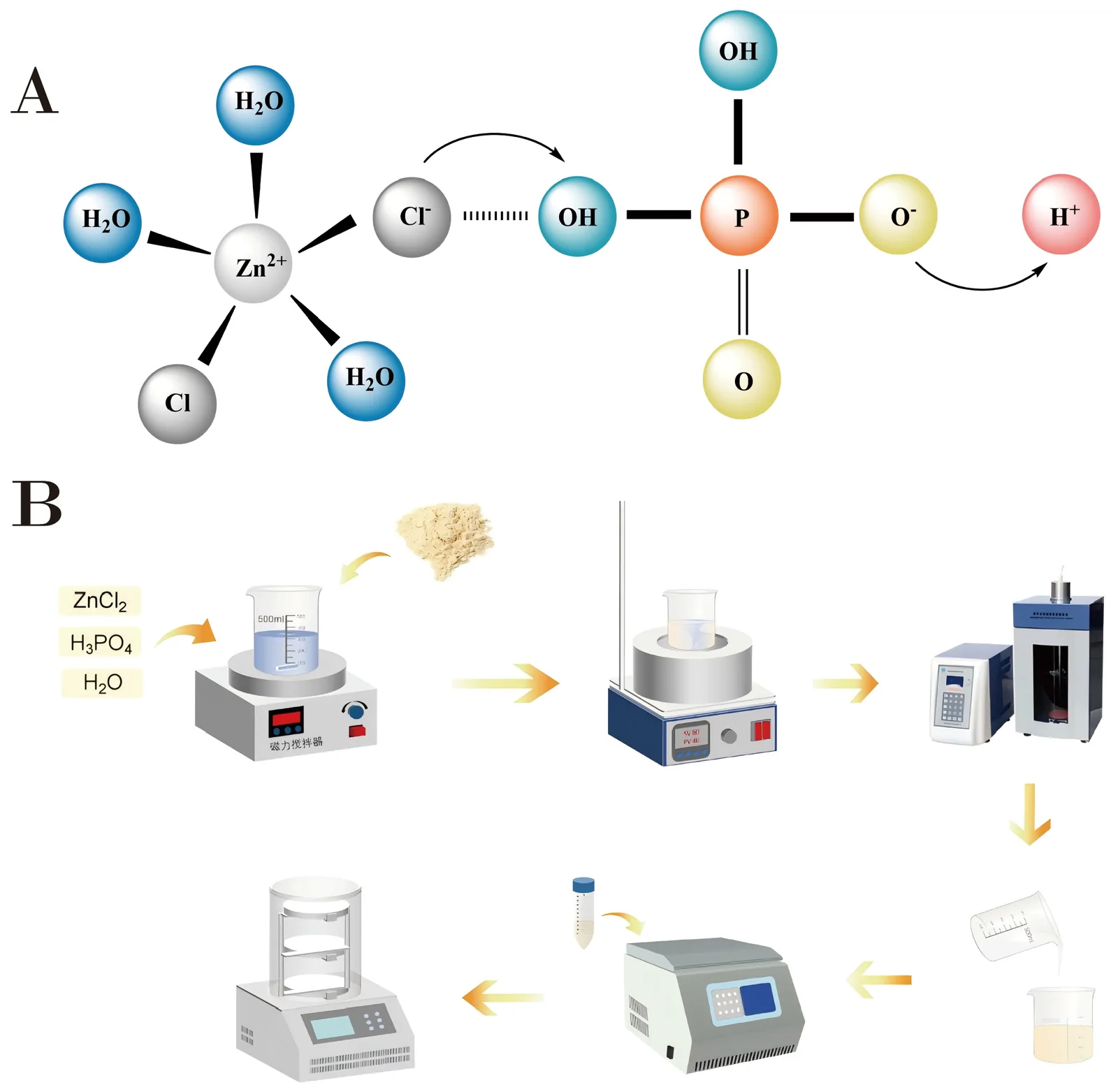
Preparation of DES and Pretreatment of SPI (**A**): Schematic diagram of low-melting-point solvent synthesis; (**B**): Flowchart of soybean protein pretreatment process.

The pretreatment was conducted using a ternary DES system consisting of ZnCl_2_, H_3_PO_4_, and H_2_O, with the molar ratio defined as ZnCl:H_3_PO_4_: H_2_O = 1:x:y, where x represents the molar multiple of phosphoric acid relative to ZnCl_2_, and y stands for the molar multiple of water relative to ZnCl_2_. According to the single-factor optimization results presented in Section 3.1, the optimal fixed molar ratio of DES for SPI pretreatment is confirmed as ZnCl_2_:H_3_PO_4_: H_2_O = 1:0.6:2.5 (i.e., x = 0.6, y = 2.5). In this hydrated DES, ZnCl_2_ is the predominant component (~57 wt%) and H_3_PO_4_ provides Brønsted acidity, while water (~19 wt%, ~61 mol%) is included to lower viscosity, promote hydration and facilitate mass transfer. It should be noted that the solid–liquid phase diagram (eutectic point) of this hydrated system was not determined by DSC in this study, so the DES designation is based on the formulation and preparation procedure rather than on a measured eutectic point. As a widely recognized green medium for biomass modification [33], the target DES was prepared by mixing the three raw materials at the optimized proportion and magnetically stirring on a DF-101S thermostatic heating magnetic stirrer (Shanghai Lichen Bangxi Instrument Technology Co., Ltd., Shanghai, China) at 60 °C for 5 h until a fully transparent, homogeneous liquid was obtained, as illustrated in Figure 1A [34].

SPI powder was blended with the prefabricated DES at a solid–liquid ratio of 1:20 (*w*/*v*). The mixture was continuously stirred in an oil bath held at a constant reaction temperature of 60 °C for a fixed reaction duration of 5 h, using a DF-101S constant-temperature heating magnetic stirrer (Shanghai Lichen Bangxi Instrument Technology Co., Ltd., Shanghai, China). After thermal incubation, ultrasonic treatment was implemented using a JY92-IIDN ultrasonic cell disruptor (Ningbo Xinzhi Biotechnology Co., Ltd., Ningbo, China) with standardized operational parameters: ultrasonic power of 300 W, a frequency of 20 kHz, an amplitude of 60%, intermittent mode (work 3 s, pause 2 s), and a total ultrasonication time of 15 min, to further disassemble the higher-order biopolymer architectures [35]. After the ultrasonic reaction finished, the mixed system was diluted with distilled water and stored at 4 °C for 12 h. During water dilution and low-temperature standing, the DES-dissolved protein re-aggregated and precipitated; the recovered precipitate was therefore the structurally modified (disintegrated and re-aggregated) SPI used for subsequent film preparation. The suspension was centrifuged at 7000 r∙min^−1^ for 15 min via a DD5 high-speed desktop centrifuge (Hunan Kaida Scientific Instrument Co., Ltd., Changsha, China) to separate the precipitate and supernatant [36].

The resulting precipitate was washed three times with distilled water, with the supernatant collected and discarded via centrifugation at 7000 r∙min^−1^ for 15 min after each wash cycle. The purified precipitate was subsequently freeze-dried with a BK-FD12T vacuum freeze dryer (Shandong Boke Biotechnology Industry Co., Ltd., Jinan, China) to obtain pretreated SPI, as shown in Figure 1B.

To establish the optimal pretreatment protocol, a systematic single-factor experimental design was adopted to evaluate the effects of key parameters on SPI solubility, including the H_3_PO_4_ molar ratio (0.2, 0.4, 0.6, 0.8, 1.0), H_2_O molar ratio (2.0, 2.5, 3.0, 3.5, 4.0), reaction time (1, 2, 3, 4, 5 h), reaction temperature (40 °C, 60 °C, 80 °C, 100 °C, 120 °C), and ultrasonication duration (0, 5, 10, 15, 20 min) [37].

The degree of SPI dissolution was assessed by determining the soluble-protein concentration in the centrifugal supernatant via the Coomassie brilliant blue G-250 colorimetric assay (BSA calibration); this value reflects the protein dissolution/disintegration induced by DES–ultrasound and was used as the response for pretreatment optimization.

#### 2.2.2. Characterization of Pretreated SPI

##### Particle Size Distribution

The supernatant of pretreated SPI obtained after centrifugation was subjected to serial dilution with pure deionized water: 1 mL of raw supernatant was mixed with 49 mL of deionized water to achieve a 50-fold dilution, and the final SPI concentration of the diluted suspension was controlled at approximately 0.08 mg∙mL^−1^. Subsequently, the average particle size and particle size distribution were measured using a NANOTRAC WAVE II nanoparticle size analyzer (Microtrac, Montgomeryville, PA, USA) based on dynamic light scattering (DLS) principles.

##### Fourier Transform Infrared (FTIR) Spectroscopy

FTIR spectroscopy was applied to characterize the functional groups and chemical structure of both native SPI and DES-pretreated SPI samples. Prior to testing, dried SPI samples were ground into fine powder and thoroughly mixed with spectroscopic-grade potassium bromide (KBr) at a mass ratio of 1:100. The mixture was then pressed into transparent circular pellets under 10 MPa for 1 min. Spectra were recorded using a WQF-510A Fourier transform infrared spectrometer (Beijing Rayleigh Analytical Instrument Corporation, Beijing, China) within a wavenumber range of 4000–400 cm^−1^, with 32 scans per sample at a resolution of 4 cm^−1^.

##### X-Ray Diffraction (XRD) Analysis

XRD patterns of SPI samples were obtained using an X’Pert PRO X-ray polycrystalline diffractometer (PANalytical, Almelo, The Netherlands) with Cu Kα radiation. Tests were performed in the 2θ range of 5–60° at a scanning rate of 5°∙min^−1^ with a step size of 0.02°. The crystallinity of samples was calculated from the diffraction peak areas. The crystallinity of samples was calculated from the diffraction peak areas. Peak-area separation and integration were performed consistently for all samples.

The calculation formula is as follows:

(1) $$Crl(\%)\;=\;\frac{Ac}{Ac+Aa}\times 100\%$$

where *Ac* is the integrated area assigned to crystalline diffraction peaks and *Aa* is the integrated area of the amorphous halo.

#### 2.2.3. Preparation of SPI–CS Composite Films With or Without Antioxidant Properties

CS was dissolved in 1% (*v*/*v*) aqueous acetic acid to formulate a 1.5% (*w*/*v*) stock solution [35]. Separately, SPI was dispersed in deionized water to prepare a 2% (*w*/*v*) suspension, which was magnetically stirred at 70 °C for 30 min to induce protein denaturation before cooling to ambient temperature.

**Figure 2:**
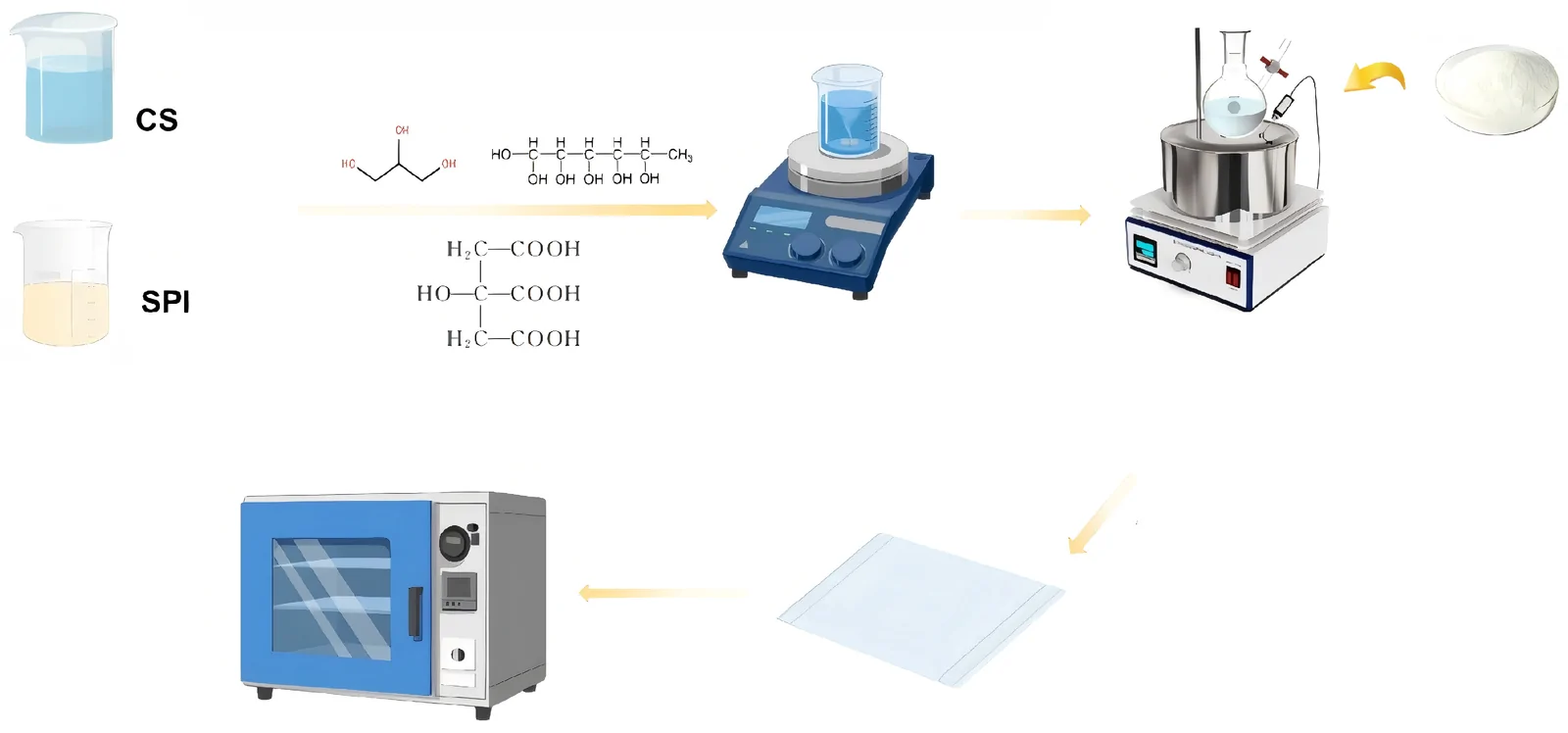
Synthesis flow chart of soybean protein–chitosan film.

The CS and SPI solutions were blended at a volume ratio of 9:1, corresponding to a dry-mass ratio of approximately 6.75:1 (CS:SPI; CS ~87.1 wt%, SPI ~12.9 wt% of the total biopolymer dry mass, based on stock concentrations of 1.5% *w*/*v* CS and 2% *w*/*v* SPI), followed by the addition of composite plasticizers accounting for 2 wt% of the total mixed system (glycerol:sorbitol:citric acid mass ratio = 2:1:0.84). The mixture pH was adjusted to 4.5 and stirred magnetically at room temperature for 2 h, then transferred to an 80 °C oil bath and heated for 30 min prior to cooling down to 40 °C. TG was incorporated primarily to catalyze ε-(γ-glutamyl)lysine isopeptide crosslinks among SPI chains, with the free primary amines on chitosan potentially acting as additional acyl acceptors [36], and the crosslinking reaction proceeded under stirring at 45 °C for 2 h. The enzyme was finally deactivated via heating for 5 min at 80 °C to acquire the base film-forming solution. The overall fabrication procedure of the dual-crosslinked SPI–CS composite films is schematically illustrated in Figure 2.

Box–Behnken design (BBD), a classic response surface methodology [37], was adopted to optimize the fabrication parameters of antioxidant-free SPI–CS films. Citric acid mass ratio (A), TG dosage (B) and film-forming temperature (C) were selected as independent variables, with tensile strength and elongation at break set as the two response values.

To fabricate antioxidant composite films, Car or GA with mass fractions ranging from 0.5% to 2.5% were separately mixed into the optimized base film solution after it cooled to room temperature, followed by thorough stirring to achieve uniform dispersion.

All film-forming solutions underwent vacuum degassing and were quantitatively cast onto 10 cm × 10 cm square acrylic plates (plate thickness: 1.5 cm). A volume of 7.0 ± 0.1 mL of film-forming solution was uniformly poured onto each plate to obtain target-thickness films. Subsequently, the cast plates were dried in a DHG-9075-III electric blast drying oven (Shanghai Yiheng Technology Co., Ltd., Shanghai, China) at 65 °C. After stripping from the substrates, five random points on each film were measured with a digital thickness meter (precision = 0.001 mm). The average thickness of all the film samples was maintained at 0.12 ± 0.01 mm. The thickness of each test specimen was recorded before mechanical and contact angle measurements to exclude thickness-induced data deviation. Prior to characterization, all films were equilibrated for 3 h in a desiccator filled with saturated magnesium nitrate solution (≈53% RH) [38]. The full names and abbreviations of all film specimens are summarized in Table 1.

#### 2.2.4. Characterization of SPI–CS Composite Films

##### Mechanical Properties

Tensile strength and elongation at break were tested following the Chinese national standard GB/T 1040.3-2006 [39] using an MWD-10B universal mechanical testing machine (Xinsansi Testing Instrument Co., Ltd., Jinan, China). Films were cut into 15 × 100 mm rectangular specimens, and the thickness was measured before testing. The initial grip distance was set to 60 mm, and the tensile speed was 5 mm∙min^−1^. The tensile force and elongation at fracture were recorded, and all tests were performed in triplicate with the mean value reported.

##### Water Resistance

(1) Solubility Determination

Film samples were cut into square specimens (20 × 20 mm), and approximately 1 g of film samples was accurately weighed using a UTP-313 electronic analytical balance (Shanghai Yukang Teaching Instrument Equipment Co., Ltd., Shanghai, China). The samples were immersed in 50 mL of distilled water and stirred at ambient temperature for 24 h. The insoluble residues were subsequently collected, dried to constant weight, and weighed. Each sample was tested in triplicate [40].

The calculation formula is as follows:

(2) $$\mathrm{Solubility}\;(\%)\;=\frac{W_{1}-W_{2}}{W_{1}}\times 100\%$$

where $W_{1}$ is the initial mass of the test film sample (g) and $W_{2}$ is the constant dry mass of the insoluble residue after water immersion (g).

(2) Water Contact Angle Analysis

The water contact angle was measured on the air-facing side of dried films using a DSA30S water contact angle meter (Guangdong Shengding Co., Ltd., Dongguan, China) to evaluate surface hydrophilicity/hydrophobicity. Deionized water (1 μL per droplet) was used as the test liquid, and the angle was read at a fixed 10 s after droplet deposition (quasi-equilibrium). Each sample was tested at three different positions, and the average value was recorded.

##### Color Analysis

The surface color of the film, specifically L* (lightness), a* (red-green value), and b* (yellow-blue value), was assessed using a CR-410 color difference meter (Konica Minolta, Tokyo, Japan). Each film underwent five parallel measurements. The total color distinction (ΔE) was computed using the subsequent formula:

(3) $$\Delta E\;=\sqrt{\;(L^{*}\;-\;L{)}^{2}\;+\;(a^{*}\;-\;a{)}^{2}\;+\;(b^{*}\;-\;b{)}^{2}}$$

##### Morphological and Structural Characterization

(1) Scanning Electron Microscopy (SEM)

The surface microstructure of the films was observed using an SU8600 scanning electron microscope (HITACHI Science and Technology Co., Ltd., Tokyo, Japan) at an accelerating voltage of 3.0 kV. All film samples were sputter-coated with a thin gold layer before observation to improve electrical conductivity.

(2) FTIR Spectroscopy of Composite Films

FTIR spectroscopy was performed to analyze the intermolecular interactions between film components, using uncrosslinked SPI–CS film and citric acid/TG dual-crosslinked SPI–CS film as test samples. Dried film samples were ground into fine powder and and thoroughly mixed with spectroscopic-grade KBr at a mass ratio of 1:100, then pressed into transparent pellets under 10 MPa for 1 min. Spectra were collected using a WQF-510A Fourier-transform infrared spectrometer (Beijing Rayleigh Analytical Instrument Corporation, Beijing, China) over the wavenumber range of 4000–400 cm^−1^, with 32 scans per sample at a resolution of 4 cm^−1^.

##### Thermogravimetric (TG) Analysis

The thermal stability of films was evaluated using a STA 449F3 simultaneous thermogravimetric analyzer (NETZSCH Instrument Manufacturing Co., Ltd., Selb, Germany). Samples (5–10 mg) were placed in an alumina crucible and heated from 30 °C to 600 °C at a heating rate of 10 °C∙min^−1^ under a nitrogen atmosphere (50 mL∙min^−1^). The mass-loss curve and derivative thermogravimetry (DTG) curve were recorded for analysis.

##### Antioxidant Activity Assay

The antioxidant capacity of films was evaluated via the DPPH free radical-scavenging assay. Film samples (0.5 g) were immersed in 30 mL of deionized water and soaked for 24 h to obtain the film extract. Then, 3 mL of the extract was mixed with 1 mL of DPPH solution (0.1 mmol·L^−1^ in absolute ethanol), vortexed thoroughly, and incubated in the dark at room temperature for 30 min. The absorbance at 517 nm was measured using a UV754N UV-visible spectrophotometer (Shanghai Youke Instrument Co., Ltd., Shanghai, China). Each sample was tested in triplicate. The calculation formula is as follows:

Where A_DPPH_ is the absorbance of the DPPH solution (control) and A_0_ is the absorbance of the sample solution.

(4) $$\mathrm{Scavenging}\;\mathrm{rate}\;(\%)=\frac{A_{DPPH}-A_{0}}{A_{DPPH}}\times 100\%$$

### 2.3. Cherry Tomato Preservation Assay

Uniformly sized, mature cherry tomatoes with intact peel, free of mechanical wounds and lesions, were screened as experimental samples. The fruits were lightly rinsed with distilled water to eliminate surface contaminants and air-dried naturally at ambient temperature. The qualified fruits were randomly assigned to four treatment groups, each containing 30 tomatoes separated into three biological replicates (10 fruits per replicate). Fruits of each group were transferred to food-grade rigid polypropylene (PP) plastic boxes (inner dimensions: 15 × 10 × 8 cm), whose openings were fully sealed with the corresponding composite films using food-grade silicone sealant along the rim: pure SPI–CS film, SPI–Car single-doped film, SPI–GA single-doped film, and dual Car/GA co-loaded SPI–CS film. The effective film area covering each box opening was 15 × 10 cm = 150 cm^2^ (0.015 m^2^), and the internal box volume was approximately 1.2 L; after loading 10 cherry tomatoes (total fruit mass ≈ 180 g), the residual headspace was about 1.0 L. All packaged samples were stored for 12 days under controlled conditions of 25 ± 2 °C and 50–55% relative humidity. The relative humidity inside each storage chamber was maintained at 50–55% using a saturated magnesium nitrate (Mg(NO_3_)_2_) solution placed at the bottom of the chamber. A calibrated digital temperature-humidity logger was placed inside each chamber to continuously monitor RH and temperature throughout the 12-day storage period. Sampling and index detection were implemented at 2-day intervals. At each sampling time, three fruits were randomly picked from every biological replicate for measurement, and all assays were conducted in triplicate.

#### 2.3.1. Ascorbic Acid (Vc) Content Determination

Vc content in cherry tomatoes was determined via the 2,6-dichlorophenolindophenol titration method. Briefly, 10 g of cherry tomato sample was rapidly frozen in liquid nitrogen, ground into powder, and transferred to a 100 mL volumetric flask. The volume was fixed with 2% oxalic acid solution, shaken well, left to stand for 30 min, and then filtered. Then, 10 mL of the filtrate was titrated with 2,6-dichlorophenolindophenol solution (0.2 g·L^−1^ in water, freshly standardized against a standard L-ascorbic acid solution to determine the dye titer ρ) until a pink color persisted for 30 s (endpoint). The consumed volume of titrant was recorded, and each group was tested three times. The Vc content was calculated according to Formula (5):

(5) $$\mathrm{Vc}(\frac{\mathrm{mg}}{100\;g})\;=\;\frac{V\;-(V_{1}\;-V_{2})\;\times \;\rho }{\rho V^{\prime }\times m}\times 100\%$$

where: V = total volume of cherry tomato extract, mL; V_1_= volume of 2,6-dichlorophenolindophenol consumed in blank titration, mL; V_2_ = volume of 2,6-dichlorophenolindophenol consumed for sample filtrate titration, mL; ρ = mass of ascorbic acid equivalent to 1 mL 2,6-dichlorophenolindophenol solution, mg∙mL^−1^; V’ = volume of filtrate taken for titration, mL; and m = mass of cherry tomato sample, g.

#### 2.3.2. Weight Loss Rate Determination

Cherry tomatoes were separated into four film-sealed groups and stored at 25 ± 2 °C, 50–55% RH for 12 d. Mass measurements were conducted every 2 d (Day 0, 2, 4, 6, 8, 10, 12). At each sampling, fruits from every replicate were quickly weighed on a UTP-313 electronic analytical balance (Shanghai Yukang Teaching Instrument Equipment Co., Ltd., Shanghai, China) to prevent water evaporation. Since the PP box walls and the silicone-sealed rim are essentially impermeable to water vapor, the measured weight loss predominantly reflects water vapor transpired from the fruit and diffused through the film lid into the surrounding atmosphere (50–55% RH); it thus provides an in-package indication of water-vapor transmission under actual storage conditions, rather than a standardized WVTR measurement. The weight loss rate was calculated according to Formula (6):

(6) $$Weight\;Loss\;Rate(\%)\;=\;\frac{m1-m2}{m1}\times 100\%$$

where m1 is the initial mass of cherry tomatoes on day 0 of storage (g) and m2 is the mass of cherry tomatoes on day t of storage (g).

#### 2.3.3. Rot Rate Determination

Rot area is classified into four grades:

Grade 0—No rot area;

Grade 1—Rot area accounts for less than 10% of the total fruit surface area;

Grade 2—Rot area accounts for 10% to 30% of the total fruit surface area;

Grade 3—Rot area accounts for more than 30% of the total fruit surface area.

The rot rate is calculated using Formula (7), a common index for evaluating fruit decay:

(7) $$\mathrm{Rot}\;\mathrm{Rate}\;(\%)\;=\;\frac{\Sigma (Rot\;Grade\times Number\;of\;Cherry\;Tomatoes\;in\;That\;Grade)}{Highest\;Rot\;Grade\times Total\;Number\;of\;Cherry\;Tomatoes}$$

#### 2.3.4. Sensory Evaluation

Quantitative sensory analysis was carried out to track variations in cherry tomato appearance, peel color, pulp texture and epidermal shriveling over the 12-day storage cycle. A validated 9-point structured hedonic scale was adopted [41,42], with the unified scoring criteria specified below:

9 points: Excellent—fully intact fruit, bright red peel, firm pulp, no decay;

8 points: Very good—almost fully intact fruit, negligible epidermal wrinkling, bright red peel, firm pulp;

7 points: Good—mild epidermal shriveling, slightly dull red color, moderately firm pulp;

6 points: Fairly good—noticeable but mild epidermal shriveling, slightly dull red color, moderately softened pulp without obvious decay;

5 points: Fair—moderate epidermal shriveling, dark-red discoloration, obviously softened pulp;

4 points: Poor—noticeable peel shrinkage, dark-red discoloration, obvious pulp softening;

3 points: Very poor—severe shriveling, obvious browning, partial pulp deterioration;

2 points: Extremely poor—severe shriveling, obvious browning, partial pulp deterioration before full decay;

1 point: Unacceptable—complete decay, severe browning, inedible.

The sensory panel consisted of 10 trained evaluators (5 males, 5 females, aged 22–30). All panelists attended standardized pre-test training, which covered scoring rules, deterioration feature recognition and blind assessment procedures to reduce subjective deviation. All fruit samples were randomly coded to mask film treatment groups during evaluation. Sensory scoring was performed in ten replicates for each packaging group at 2-day sampling intervals (Day 0, 2, 4, 6, 8, 10, 12). Mean scores paired with standard deviation were calculated for inter-group statistical comparison. Meanwhile, digital photographs of cherry tomatoes were recorded synchronously to provide intuitive visual support for the quantitative sensory results.

### 2.4. Data Processing

All independent experiments were carried out in three biological replicates, and experimental data were expressed as mean ± standard deviation (SD). Response surface methodology (RSM) based on Box–Behnken design (BBD) was adopted to optimize the film fabrication process, and all RSM data processing, regression modeling and statistical significance evaluation were completed using Design Expert 13.1 software; OriginPro® 2022 software (OriginLab Corporation, Northampton, MA, USA). was utilized for plotting all histograms, spectral curves and response surface contour graphs.

For comparisons among treatments at a given condition or storage time, data were analyzed by one-way ANOVA followed by Tukey HSD post hoc test (α = 0.05). Distinct lowercase letters denote statistically significant differences between treatments.

## 3. Results

### 3.1. Optimization of DES Pretreatment Conditions for SPI

**Figure 3:**
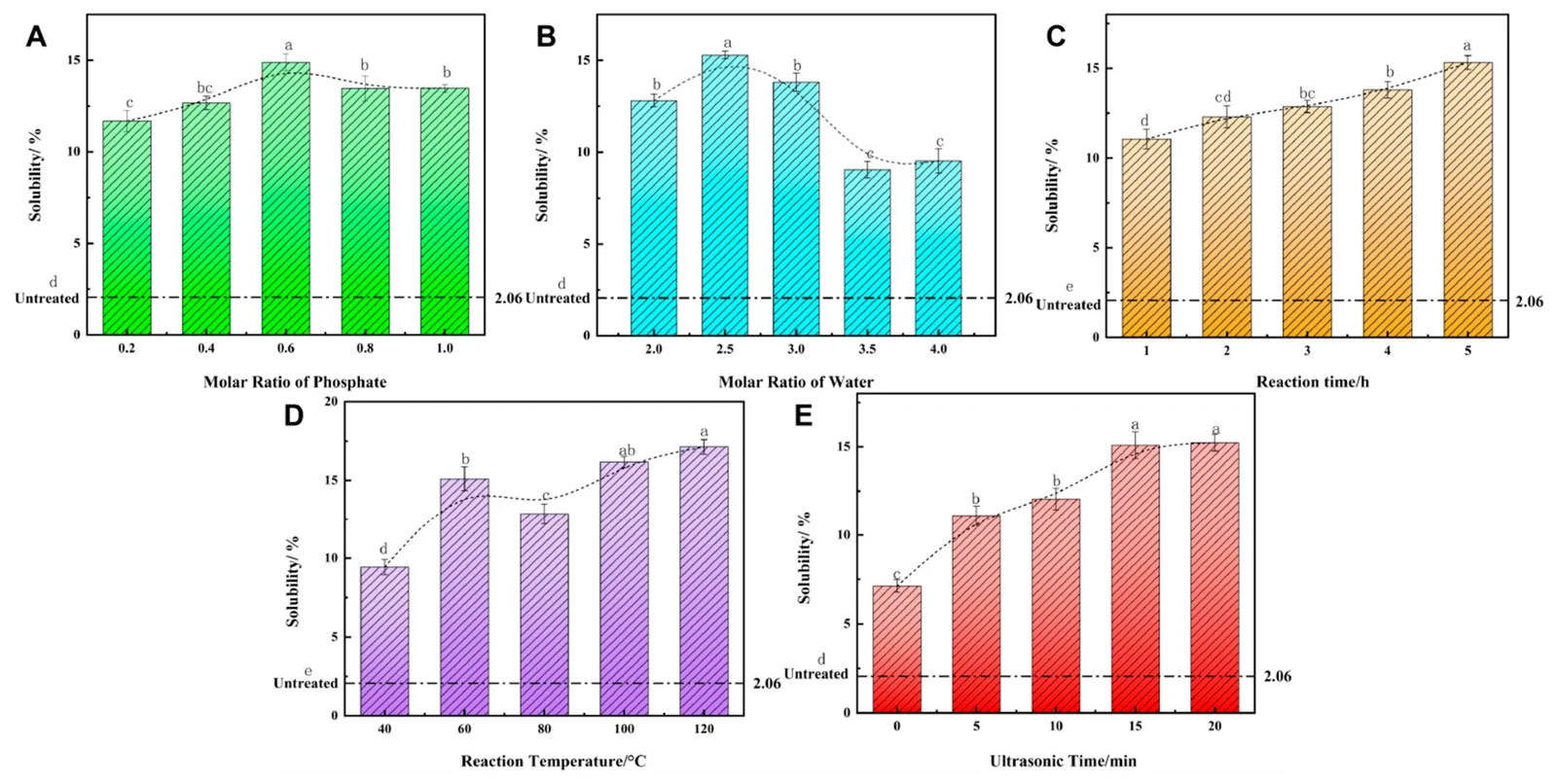
Effects of different pretreatment conditions on soluble-protein content in the supernatant. (**A**) Phosphoric-acid molar ratio; (**B**) Molar ratio of water; (**C**) Reaction time; (**D**) Reaction temperature; (**E**) Ultrasonication duration. Different lowercase letters indicate significant differences (*p* < 0.05, one-way ANOVA followed by Tukey’s HSD test).

As shown in Figure 3, five independent single-factor experiments were carried out to investigate the influences of five DES pretreatment parameters on the soluble-protein content in the supernatant, with one variable altered each time and the remaining three parameters kept constant:

1. Phosphoric-acid molar ratio gradient (0.2, 0.4, 0.6, 0.8, 1.0): Fixed ZnCl_2_:H_2_O molar ratio = 1:2.5, reaction time = 5 h, reaction temperature = 60 °C, ultrasonication duration = 15 min;
2. Water molar ratio gradient (2.0, 2.5, 3.0, 3.5, 4.0): Fixed ZnCl_2_:H_3_PO_4_ molar ratio = 1:0.6, reaction time = 5 h, reaction temperature = 60 °C, ultrasonication duration = 15 min;
3. Reaction temperature gradient (40, 60, 80, 100, 120 °C): Fixed ZnCl_2_:H_3_PO_4_:H_2_O molar ratio = 1:0.6:2.5, reaction time = 5 h, ultrasonication duration = 15 min;
4. Reaction time gradient (1, 2, 3, 4, 5 h): Fixed ZnCl_2_:H_3_PO_4_:H_2_O molar ratio = 1:0.6:2.5, reaction temperature = 60 °C, ultrasonication duration = 15 min;
5. Ultrasonication duration gradient (0, 5, 10, 15, 20 min): Fixed ZnCl_2_:H_3_PO_4_:H_2_O molar ratio = 1:0.6:2.5, reaction time = 5 h, reaction temperature = 60 °C.

The soluble-protein content in the supernatant reached its maximum at an H_3_PO_4_ molar ratio of 0.6. At this ratio, the moderate acidity of the DES system effectively disrupted the hydrogen-bond network within SPI without triggering excessive protein aggregation induced by overly strong acidity. When the H_2_O molar ratio was fixed at 2.5 for this set of tests, the DES maintained balanced viscosity, offering suitable fluidity and solvation capacity to disperse and dissolve SPI fully. This content peaked at a reaction time of 5 h and a temperature of 60 °C, which provided sufficient contact between DES and SPI to break tertiary and quaternary protein structures and release embedded active functional groups. A 15-min ultrasonic treatment yielded the highest soluble-protein content as cavitation and mechanical vibration fragmented large SPI aggregates. The solubility data shown in Figure 3 were calculated from the soluble-protein content of supernatants determined by the Coomassie brilliant blue G-250 assay, which quantitatively reflects the dissolution completeness of SPI under different DES–ultrasound parameters. In summary, the optimal SPI pretreatment conditions were determined as ZnCl_2_:H_3_PO_4_:H_2_O = 1:0.6:2.5, reaction time = 5 h, reaction temperature = 60 °C, and ultrasonication duration = 15 min.

### 3.2. Structural Characterization of Pretreated SPI

**Figure 4:**
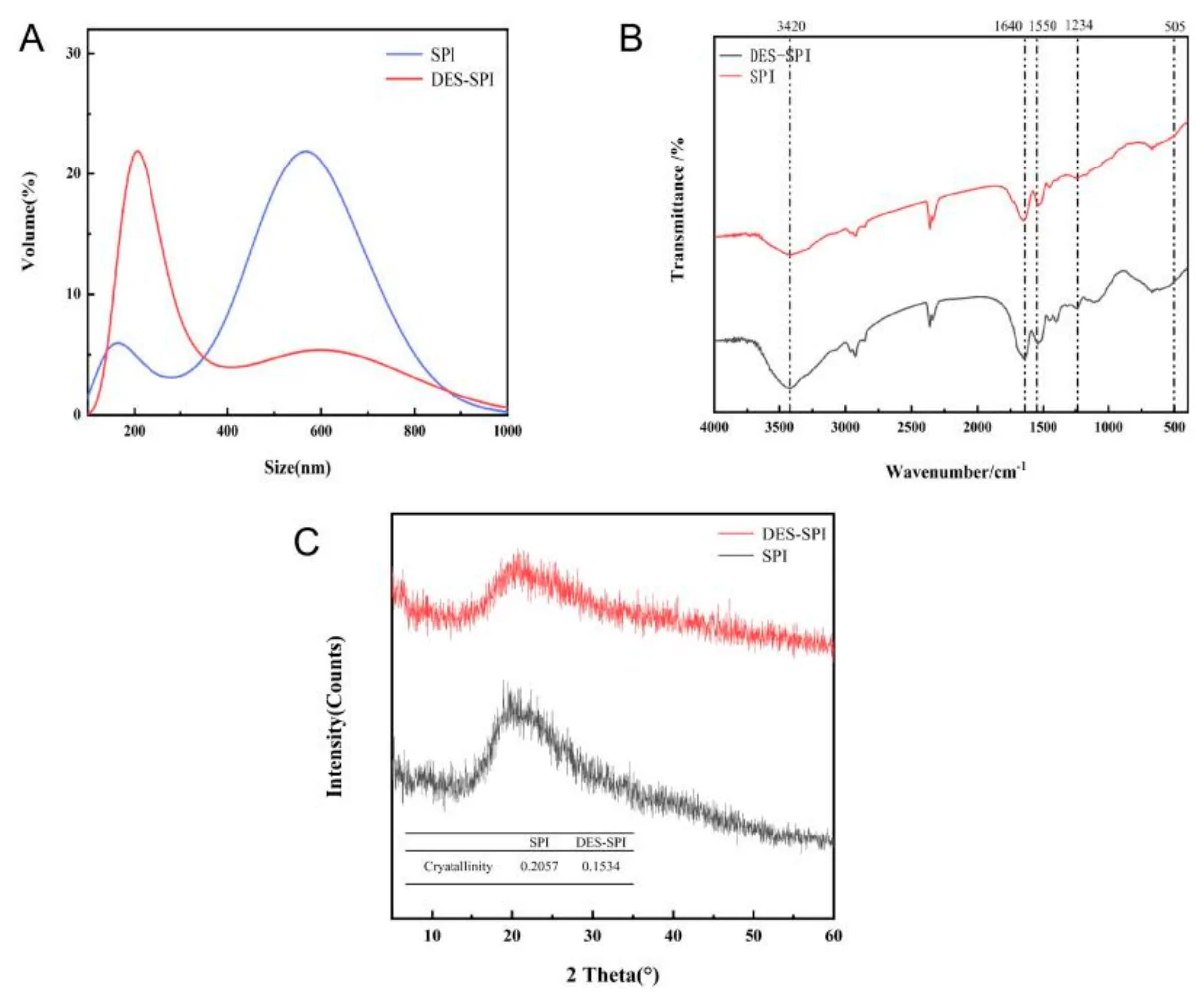
Structural characterization of native SPI and DES-pretreated SPI. (**A**) Particle size distribution curves; (**B**) FTIR spectra; (**C**) XRD patterns.

Figure 4A displays the particle size distribution curves of native and DES-treated SPI. Both samples present two peaks within 200–1000 nm that differ in shape, position and intensity. After ultrasonic-assisted DES pretreatment, the average SPI particle size decreased from 408 nm: the large peak near 600 nm weakened and shifted to smaller sizes, while the ~200 nm small peak became more prominent. Acidic DES combined with ultrasound breaks SPI’s quaternary structure, hydrogen bonds and hydrophobic interactions to deagglomerate large protein clusters; the elevated surface charge of fragmented protein generates electrostatic repulsion to prevent re-aggregation.

Figure 4B presents FTIR spectra of untreated and DES-modified SPI. The spectrum of native SPI exhibited the characteristic absorption bands of soy protein: a broad band centered at 3420 cm^−1^ attributed to O–H and N–H stretching vibrations, absorption bands at 2960–2920 cm^−1^ corresponding to asymmetric and symmetric C–H stretching of methylene groups, the amide I band at approximately 1654 cm^−1^ (mainly C=O stretching), the amide II band at approximately 1539 cm^−1^ (N–H bending coupled with C–N stretching), the amide III band around 1234 cm^−1^, and a band at 505–540 cm^−1^ related to disulfide (S–S) bonds [43]. After DES-ultrasonic pretreatment, the intensities of the O–H/N–H stretching band at 3420 cm^−1^ and the disulfide band at 505–540 cm^−1^ were notably attenuated, indicating the dissociation of hydrogen bonds and cleavage of partial disulfide linkages within the protein aggregates. Meanwhile, the positions and relative intensities of the amide I, II and III bands remained essentially unchanged, and no new absorption bands or phosphorylation/esterification characteristic signals were detected. These results confirm that DES-ultrasonic treatment disrupted high-order protein structures and intermolecular interactions without damaging the primary polypeptide chain, in line with the reported retention of the Amide I–III bands in soy protein after NADES processing and recovery [44].

Figure 4C shows XRD patterns of raw and pretreated SPI. Consistent with the predominantly amorphous nature of soy protein, the XRD patterns display broad, diffuse halos without well-defined sharp crystalline peaks; the two characteristic diffraction features at 2θ ≈ 9° and 2θ ≈ 20° correspond to the ordered α-helix (crystalline region I) and β-sheet (crystalline region II) domains, respectively, in agreement with previous reports on SPI diffraction patterns [45,46]. To compare the relative content of ordered domains between samples, the apparent crystallinity was estimated using the standard peak-area separation method. After DES-ultrasonic pretreatment, both diffraction peaks were weakened, particularly the β-sheet peak at 2θ = 20°, which was accompanied by a slight broadening of the peaks, and the estimated relative ordered-domain content decreased from 20.57% to 15.34%. Although these values should be regarded as semi-quantitative indicators of ordered-structure content rather than absolute crystallinity, the consistent decrease indicates the breakdown of intermolecular hydrogen and disulfide bonds, which reduces ordered molecular stacking and partially destroys both ordered regions of SPI, while the primary polypeptide structure remains intact.

Conventional choline chloride binary DESs are the most commonly used systems in biomass processing [25,35], yet they rely on organic hydrogen-bond donors. The inorganic ZnCl_2_-H_3_PO_4_-H_2_O ternary DES developed here offers a low-cost alternative, dissociating protein aggregates through the synergistic hydration and protonation effects of metal ions and phosphoric acid. The relative decrease in ordered-domain content (~25.4%, from 20.57% to 15.34%) is larger than those reported for urea- (19.36%) and SDS-modified (11.26%) soy protein adhesives [47], whose initial crystallinity (~20.3%) was comparable to that of the native SPI in this work. Such a low-cost inorganic DES has rarely been discussed in the biomass-modification literature.

### 3.3. Mechanical Properties of Pretreated SPI Films

**Figure 5:**
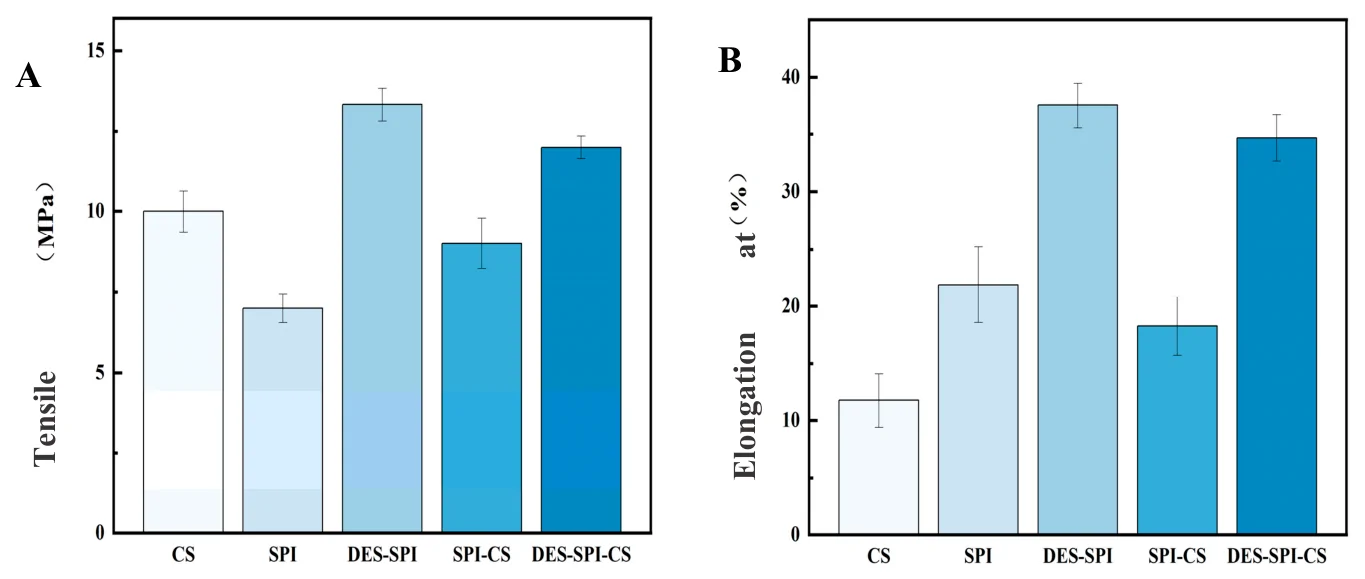
Comparison of mechanical properties of soybean protein film before and after modification. (**A**) Tensile strength; (**B**) Elongation at break.

Tensile strength and elongation at break are key indicators of the mechanical properties of packaging films—higher tensile strength means stronger stretch-failure resistance, while higher elongation at break indicates better flexibility and ductility. As shown in Figure 5A,B, films prepared with pretreated SPI exhibited significantly improved mechanical properties: tensile strength increased from 7 MPa to 13.3 MPa, and elongation at break increased from 21.9% to 37.57%. This simultaneous improvement can be attributed to the disaggregating and unfolding effect of the hydrated metal salt-based DES combined with ultrasonic treatment on SPI. The pretreatment disrupted the intramolecular hydrophobic bonds and partially destroyed the compact crystalline/aggregated structure of SPI, exposing more hydrophilic groups and releasing flexible polypeptide segments from the tightly folded globular conformation. During film formation, these unfolded SPI chains were able to establish more uniform and continuous intermolecular crosslinking with CS molecules, forming a denser yet more flexible network with fewer structural defects and stress concentration points. Consequently, the tensile strength was enhanced by the strengthened interfacial interactions and network integrity, while the elongation at break was simultaneously improved because the pre-unfolded polypeptide segments possessed higher chain mobility and flexibility. Such synergistic enhancement of both strength and ductility confirms that DES-ultrasonic pretreatment effectively optimized the molecular structure of SPI for constructing high-performance composite films.

Pure CS films are usually hard and brittle, but DES–SPI–CS composite films exhibited obvious improvements in both mechanical indicators. DES–SPI disrupted the high crystallinity and compact stacking structure of the CS matrix, and the pretreated DES–SPI possessed abundant exposed active sites to strengthen intermolecular interactions within the DES–SPI–CS system. Ultimately, compared with pure CS film, the tensile strength and elongation at break of the DES–SPI–CS film rose by 20% and 195.06%, respectively.

Such mechanical enhancement is in good agreement with the findings of earlier SPI–chitosan blend film studies, where improved interfacial compatibility between SPI and chitosan contributed to enhanced tensile and ductile performance of composite biopolymer films [48,49]. Nevertheless, our DES-ultrasonic pretreatment achieved a more prominent improvement in elongation at break than simple physical blending reported in the literature, which can be ascribed to the unfolding of compact SPI aggregates by DES–ultrasound treatment.

### 3.4. Optimization of Response Surface Design for Crosslinked Films

Three variables were selected to optimize the soy protein–chitosan composite film: citric acid mass ratio, transglutaminase (TG) addition amount, and film-forming temperature, each set at three levels.

Following the Box–Behnken central composite design principle, the experiment was designed and conducted using Design Expert 13.1.0 software. The composite film’s mechanical properties were used as response values to screen the optimal variable combination for film preparation, with the variable level coding table shown in Table 2.

This study adopted the Box–Behnken response surface model. By analyzing the changing trends of the central point, spherical surface, and central point of the spherical outer tangent plane, the relationship between the response value (mechanical properties) and the interaction among factors was clarified through spherical rotation. Table 3 shows the experimental design and results of tensile strength and elongation at break for the modified soy protein isolate–chitosan film.

#### 3.4.1. Response Surface Interaction Analysis and Optimization (Tensile Strength)

Complete ANOVA tables, fitted quadratic regression function and residual diagnostic graphs for tensile strength are available in the Supporting Information (Table S1, Figures S1–S3). Table S1 tabulates all the key statistical metrics, including the overall model *p*-value, lack-of-fit *p*-value, coefficient of determination R^2^, adjusted R^2^, predicted R^2^, adequate precision and coefficient of variation (CV). Parallel verification trials were carried out under the optimized processing parameters to validate the predictive performance of the established regression model.

Two-dimensional contour plots and three-dimensional response surface graphs were adopted to evaluate the statistical significance of individual factors and pairwise interactions toward tensile strength (the target response). A universal judging criterion was followed: denser contour lines and steeper response surfaces correspond to more remarkable single-factor or interactive effects on tensile strength. The visual contour density reflects the magnitude of the response change; formal statistical significance is determined from the ANOVA *p*-values (Table S1) rather than from contour density alone.

**Figure 6:**
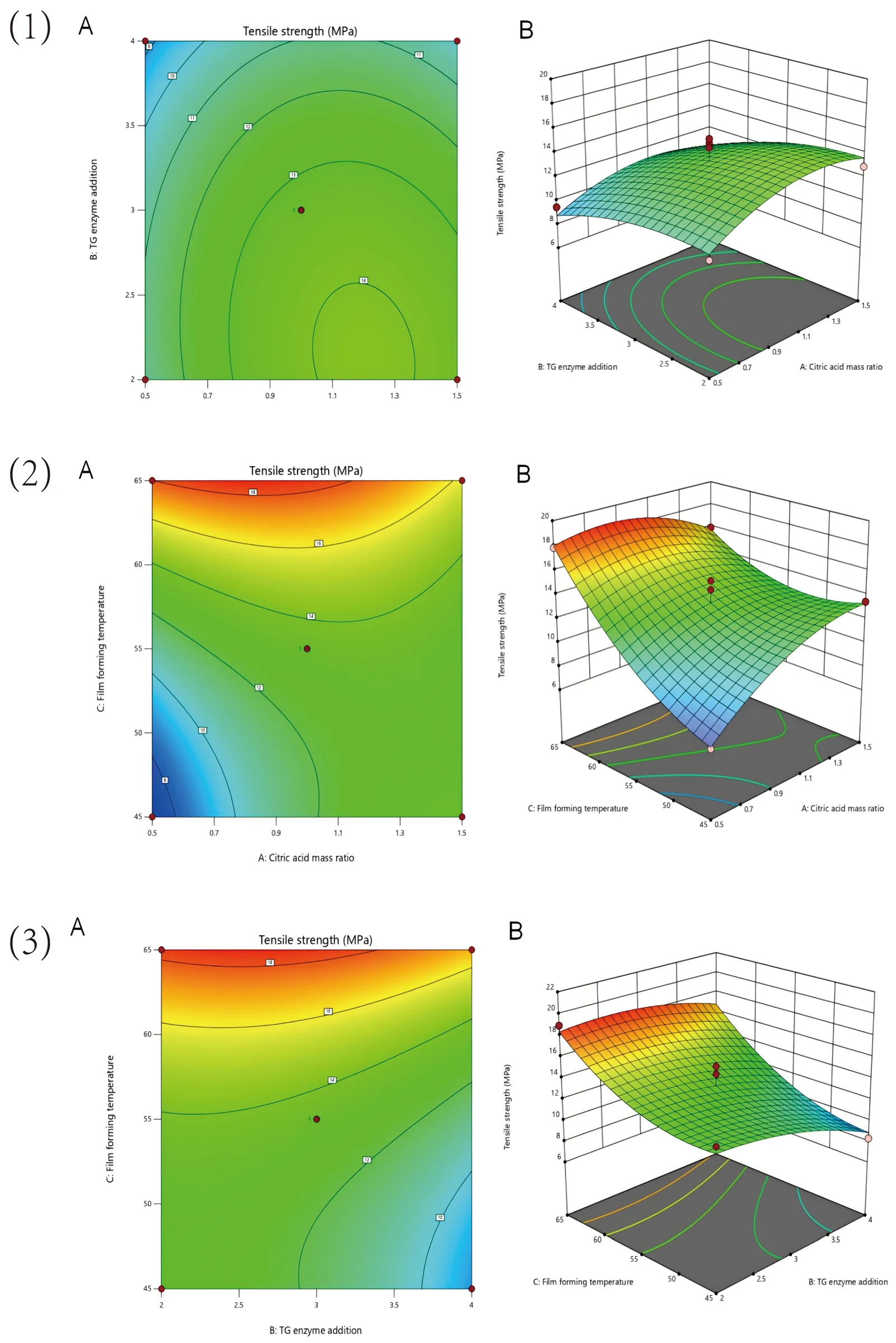
Response surface analysis of tensile strength: (**1**) Contour plot (**A**) and response surface plot (**B**) of the interaction between the mass ratio of citric acid and the addition amount of TG; (**2**) Contour plot (**A**) and response surface plot (**B**) of the interaction between citric acid mass ratio and film-formation temperature; (**3**) Contour plot (**A**) and response surface plot (**B**) of the interaction between the addition amount of TG and the film-formation temperature.

When film-forming temperature was set to the central coded level, Figure 6(1) displays the contour and 3D surface plots describing the interaction between citric acid mass ratio and TG dosage. Elliptical contour shapes signify prominent reciprocal interactions between the two variables. Further 3D surface analysis shows that tensile strength fluctuates drastically at citric acid mass ratios ranging from 0.5 to 0.9; dense contour bands across the 0.5–1.1 interval produce a steep surface gradient, indicating a pronounced trend of citric-acid influence that is borderline and did not reach formal significance in the ANOVA (*p* = 0.0615, Table S1).

Figure 6(2) illustrates the interaction between citric acid mass ratio and film-forming temperature at the central temperature level. The contour lines associated with temperature are far denser than those for citric acid, accompanied by a steeper surface slope. This demonstrates that film-forming temperature exerts a more pronounced effect on tensile strength than citric acid mass ratio within the experimental scope.

Figure 6(3) depicts the interactive relationship between TG dosage and film-forming temperature under the central temperature condition. Similarly, the contour distribution and surface gradient both confirm that film-forming temperature dominates tensile strength variation and possesses a stronger regulatory impact than TG loading.

#### 3.4.2. Response Surface Interaction Analysis and Optimization (Elongation at Break)

The full ANOVA results and quadratic regression equation for elongation at break, along with residual diagnostic graphs, are provided in the Supporting Information (Table S2, Figures S4–S6). Table S2 summarizes all the vital statistical metrics, including model significance, lack-of-fit R^2^, adjusted R^2^, predicted R^2^, adequate precision and CV. Parallel validation experiments were performed to verify the predictive accuracy of the constructed quadratic regression model.

The fitted regression equation was used to generate two-dimensional contour and three-dimensional response surface graphs, so as to quantify the significance of single factors and pairwise interactions toward elongation at break.

**Figure 7:**
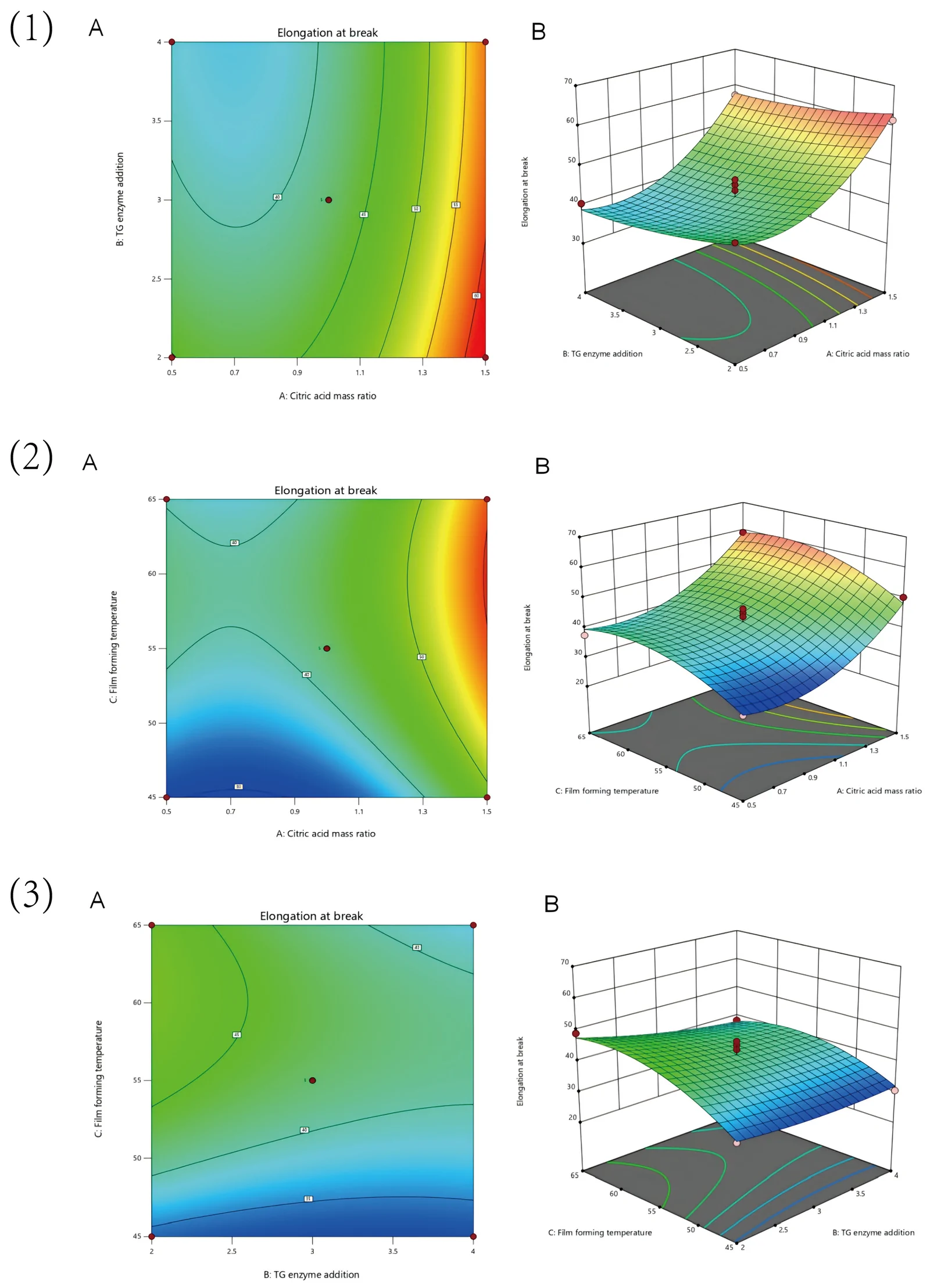
Response surface analysis of elongation at break: (**1**) Contour plot (**A**) and response surface plot (**B**) of the interaction between the mass ratio of citric acid and the addition amount of TG; (**2**) Contour plot (**A**) and response surface plot (**B**) of the interaction between citric acid mass ratio and film-formation temperature; (**3**) Contour plot (**A**) and response surface plot (**B**) of the interaction between the addition amount of TG and the film-formation temperature.

Figure 7(1) presents contour and 3D surface plots for the interaction between citric acid mass ratio and TG dosage. Elongation at break fluctuates drastically when the citric acid mass ratio falls between 0.9 and 1.5. The densely packed contour lines within this interval correspond to a steep surface gradient, which proves that citric acid proportion imposes a remarkable effect on the ductility of composite films.

Figure 7(2) depicts the interactive relationship between citric acid mass ratio and film-forming temperature. Contour lines of citric acid are much denser and paired with a steeper surface slope, demonstrating that citric acid exerts a stronger influence on elongation at break compared with film temperature within the test range.

Figure 7(3) illustrates the interaction between TG loading and film-forming temperature. In contrast to TG dosage, film-forming temperature generates tighter contour distribution and a steeper 3D surface, revealing that temperature dominates the regulation of film ductility over TG addition.

Comprehensive response surface analysis identifies the optimal fabrication parameters: citric acid mass ratio = 0.84, TG dosage = 2.60 U∙g^−1^, and film-forming temperature = 65 °C. Under this optimized system, the composite film achieves outstanding mechanical performance, with a tensile strength of 18.79 MPa and an elongation at break of 41.39%, which satisfies the fundamental property basic mechanical requirements for potential packaging applications. The optimum temperature (65 °C) lies at the upper edge of the tested range; it was retained because the fitted response plateaus near 65 °C, whereas higher drying temperatures would remove water too rapidly and induce surface crusting, blistering and uneven shrinkage that embrittle the film—consistent with the TG/DTG result that chain degradation begins only above ~150 °C.

### 3.5. Solubility and Water Contact Angle Analysis of SPI–CS Crosslinked Film

**Figure 8:**
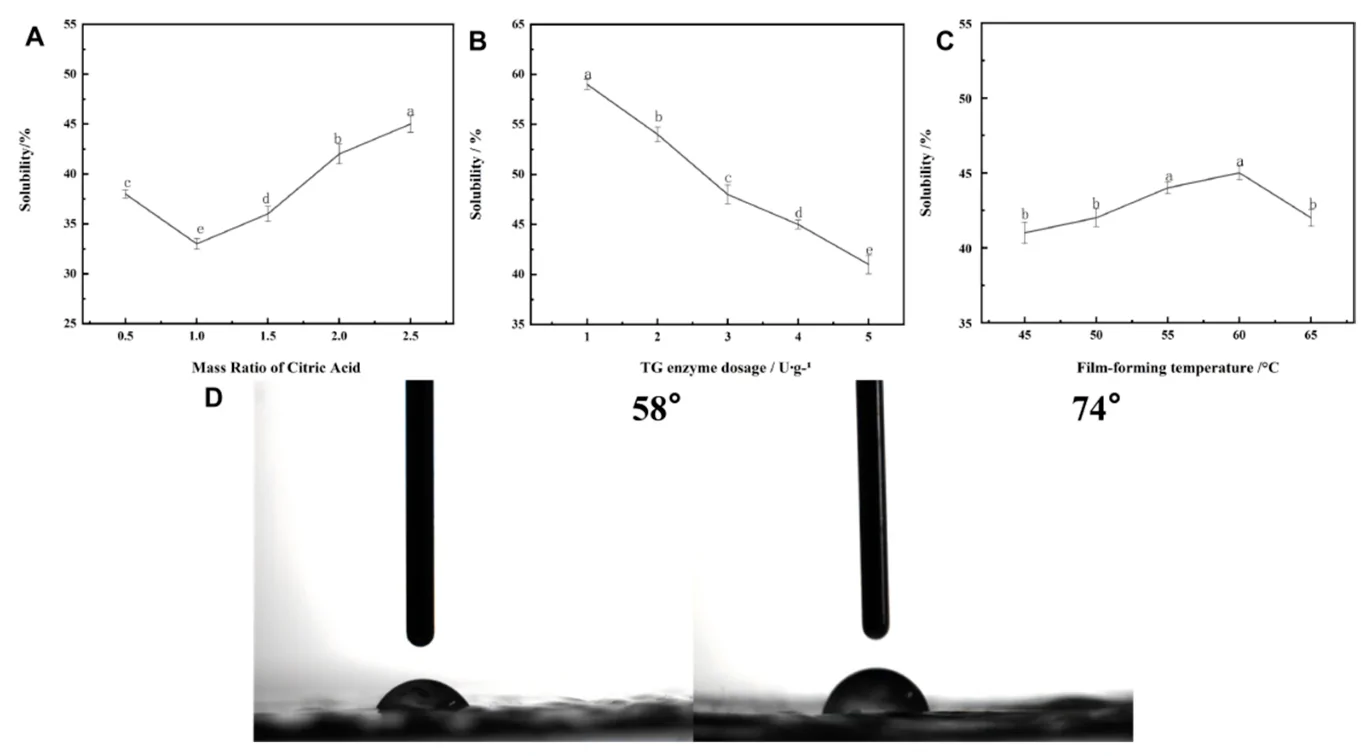
Solubility and Water Contact Angle Analysis of SPI–CS Crosslinked Films: (**A**) Citric acid mass ratio; (**B**) TG enzyme dosage; (**C**) Film-formation temperature; (**D**) Water contact angle of SPI–CS standard film versus SPI–CS modified film. Different lowercase letters indicate significant differences (*p* < 0.05, one-way ANOVA followed by Tukey HSD).

Solubility and water contact angle tests were carried out to characterize the water resistance and surface wettability of SPI–CS composite films. Four groups of single-factor trials (Figure 8A–D) were designed, wherein only one variable was adjusted each time while the baseline film formulation remained constant: CS/SPI mixing volume ratio = 9:1, total plasticizer dosage = 2 wt% (glycerol:sorbitol mass ratio = 2:1), pH = 4.5, and drying temperature = 65 °C.

For the citric acid mass gradient test shown in Figure 8A, TG dosage and film-forming temperature were fixed at 2.60 U∙g^−1^ and 65 °C, respectively, with citric acid mass levels set as 0.5, 1.0, 1.5, 2.0, and 2.5. Film solubility first decreased and then elevated as citric acid content increased. Moderate citric acid dosage introduced abundant hydrogen bonds and ionic electrostatic interactions between its multi-carboxyl groups and the amino/hydroxyl moieties of SPI and CS, constructing a dense polymer network that suppressed water penetration and dissolution. Excess free citric acid, however, retained abundant hydrophilic carboxyl groups inside the matrix, loosening the compact network and consequently increasing film solubility.

As presented in Figure 8B, the citric acid mass ratio and film-forming temperature were held constant at 0.84 and 65 °C, respectively, to investigate the influence of transglutaminase (TG) dosage ranging from 1 to 5 U∙g^−1^. Film solubility decreased monotonically with rising TG content, because transglutaminase catalyzes covalent crosslinkages between SPI polypeptide chains, enhancing intermolecular entanglement and exposing intrinsic hydrophobic segments to lower the overall hydration tendency of the film matrix [48].

For the temperature gradient experiment illustrated in Figure 8C, the citric acid mass ratio and TG dosage were fixed at 0.84 and 2.60 U∙g^−1^, respectively, with temperatures set from 45 to 65 °C at 5 °C intervals. No obvious upward or downward solubility trend was detected within this mild heating range, as moderate thermal treatment barely altered the intrinsic hydrophilic and hydrophobic chemical compositions of SPI and CS components.

The water contact angle comparison in Figure 8D was conducted between uncrosslinked SPI–CS film (without citric acid or TG) and optimally dual-crosslinked SPI–CS film (citric acid mass ratio = 0.84, TG = 2.60 U∙g^−1^) under identical preparation conditions. The water contact angle rose from 58° for the uncrosslinked sample to 74° for the crosslinked counterpart. Both angles fall below 90°, demonstrating that all films are intrinsically hydrophilic; nevertheless, the obvious angle increment reveals reduced surface hydrophilicity and improved water repellency after dual crosslinking. The synergistic hydrogen-bonded and ionic network formed by citric acid and TG rearranges polymer molecular chains and diminishes the quantity of exposed polar -OH/-NH_2_ groups on the film surface, slowing the spreading and permeation of water droplets.

In summary, the SPI–CS composite film fabricated under optimized parameters presents significantly lower solubility and milder surface hydrophilicity, indicating improved water resistance of the material.

### 3.6. Performance Analysis of SPI–CS Crosslinked Film

Table 4 shows the color values (lightness L*, red-green tone a*, yellow-blue tone b*) of four films: CS single film, deep eutectic DES–SPI single film, ordinary SPI–CS composite film, and modified crosslinked SPI–CS composite film.

Compared with the CS single film: The crosslinked SPI–CS film had a lower L* (reduced lightness) and a* (weaker red/stronger green tones), but a higher b* (stronger yellow tones), presenting a greener and yellower hue.

Compared with the DES–SPI single film: The crosslinked SPI–CS film had a higher L* and a* (higher lightness, stronger red/weaker green tones) but a lower b* (weaker yellow tones). This is due to the color neutralization effect of SPI and CS during compounding, which gives the composite film a grayish-yellow hue.

Between ordinary and crosslinked SPI–CS films: The chromaticity difference was minimal. Citric acid and TG are white powders that dissolve into colorless solutions, so their addition has a negligible impact on film chromaticity—crosslinking only causes slight changes in lightness, redness, and yellowness.

**Figure 9:**
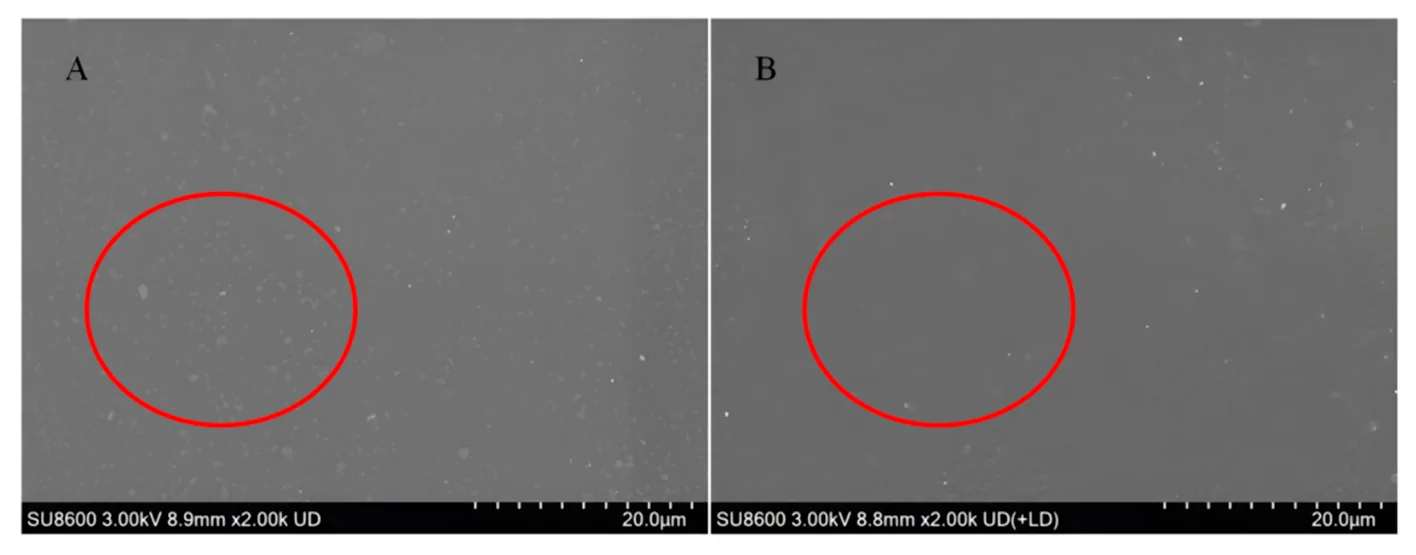
Scanning electron micrographs of (**A**) ordinary SPI–CS film and (**B**) modified SPI–CS film.

Scanning electron microscopy (SEM) analysis was performed to investigate the surface microstructure of the films and to establish the structure–performance correlation. Figure 9A presents the surface morphology of the ordinary (uncrosslinked) SPI–CS film, which exhibited a relatively smooth and continuous surface with a homogeneous dispersion of components, although a few small pores and slight roughness could be observed, indicating moderate compatibility between SPI and CS without visible phase separation. Figure 9B shows the surface morphology of the dual-crosslinked SPI–CS film. After citric acid/TG crosslinking, the film surface became noticeably denser and more compact, with reduced porosity and a slightly increased surface undulation at the microscale. The overall structural integrity was well maintained, and no cracks, voids, or phase-separation domains were detected in either sample. The densification of the modified film surface can be attributed to the covalent amide bonds formed by TG catalysis together with the ionic and hydrogen-bond bridges established by citric acid, which strengthen the intermolecular interactions between SPI and CS and enhance the interfacial compatibility of the two biopolymer matrices.

**Figure 10:**
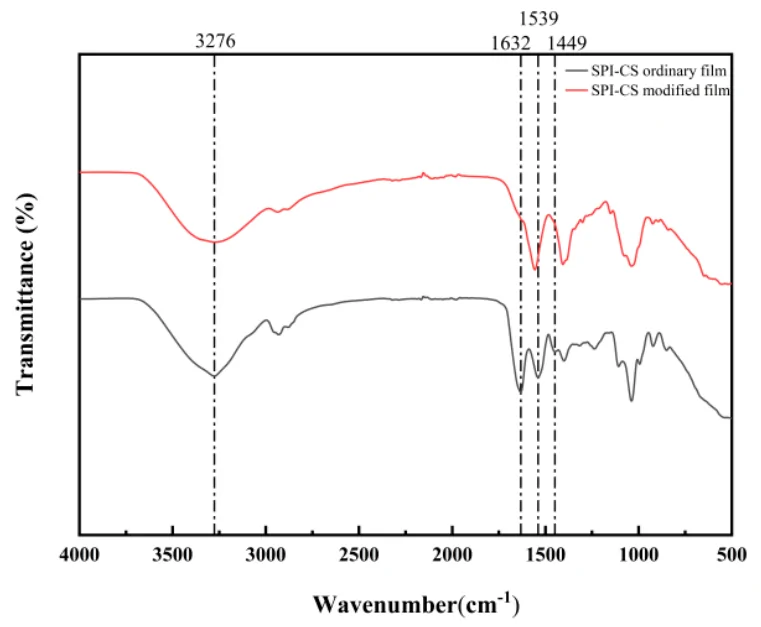
Infrared spectra of SPI–CS ordinary film and SPI–CS modified film.

FTIR spectra ranging from 4000 to 400 cm^−1^ (Figure 10) were recorded to compare intermolecular interactions within uncrosslinked and dual-crosslinked SPI–CS films, and key spectral variations are analyzed below. Notably, although the main characteristic absorption bands of the two films appear in similar wavenumber regions (marked by vertical dashed lines in Figure 10), obvious subtle peak shifts can be distinguished between uncrosslinked and dual-crosslinked samples. First, the broad O–H stretching band exhibited a red shift for the dual-crosslinked film, confirming the formation of denser intermolecular hydrogen networks triggered by TG-catalyzed crosslinking. Second, a similar red shift was observed for its N–H vibrational peaks, which is consistent with TG-catalyzed amide crosslinking, predominantly among SPI chains; whether chitosan amines also participate cannot be resolved from these spectra.

In terms of protein secondary structure, the peak at 1632 cm^−1^ is assigned to the β-sheet conformation, and the 1539 cm^−1^ band represents the typical amide II signal. Relative to the uncrosslinked SPI–CS film, the dual-crosslinked sample shows weakened absorbance at 1632 cm^−1^ together with an evident shift of the amide II peak. These spectral changes originate from the synergistic effect of citric acid and TG, rather than esterification reactions between CS amino groups and SPI hydroxyls. No ester bond formation was detectable under the mild film-forming condition—the dual-crosslinked film showed no new carbonyl band in the 1700–1750 cm^−1^ region. TG primarily catalyzes the formation of ε-(γ-glutamyl)lysine isopeptide bonds between SPI chains, while the free amines of chitosan may act as additional acyl acceptors; multi-carboxylic citric acid simultaneously establishes ionic (carboxylate) and hydrogen-bonded bridges between the two biopolymers. The combined intermolecular forces disrupt native β-sheet stacking and modify the vibrational environment of amide II, resulting in the characteristic FTIR peak shifts detected in this study.

**Figure 11:**
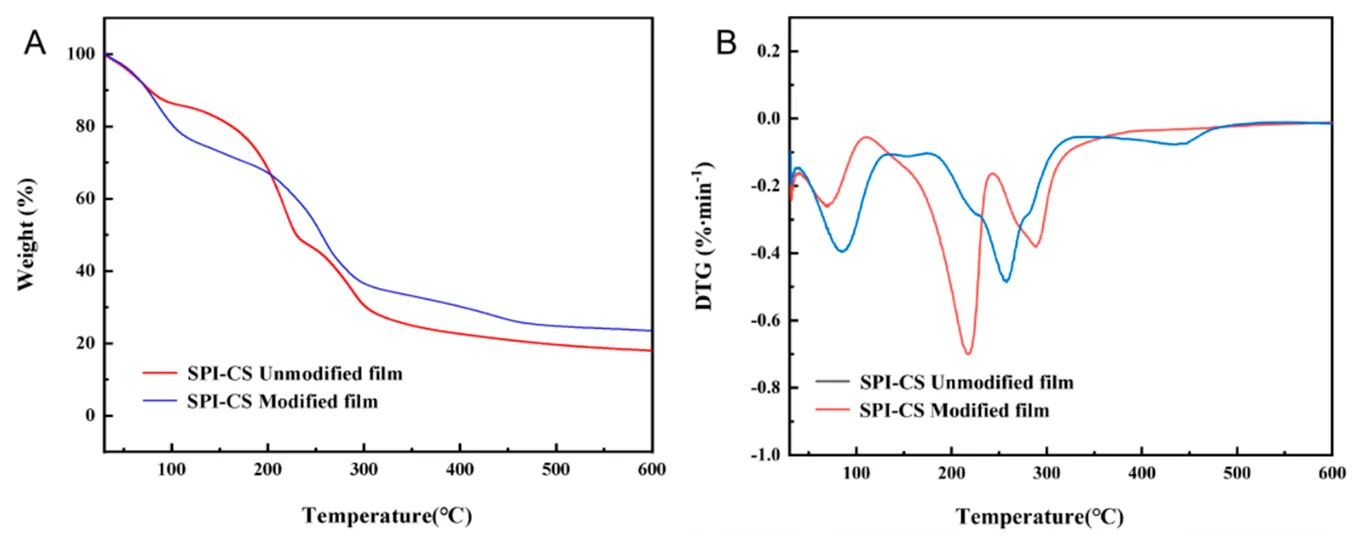
(**A**) TG and (**B**) DTG curves of SPI–CS ordinary film and SPI–CS modified film.

Thermogravimetry (TG) and derivative thermogravimetry (DTG) curves were recorded to assess thermal resistance of uncrosslinked and dual-crosslinked SPI–CS films (Figure 11). Both samples displayed three distinct mass-loss phases:

50–150 °C: Volatilization of free water and bound water trapped in polymer networks;

150–330 °C: Thermal cleavage of SPI/CS molecular chains and breakdown of intermolecular crosslink networks;

330–600 °C: Slow carbonization with negligible mass loss.

The dual-crosslinked film possessed a higher residual mass fraction (23%) than the uncrosslinked counterpart (18%). Its DTG water-loss peak shifted to higher temperature with elevated intensity, which confirms that the dense crosslink network retains more bound water inside the matrix, rather than lowering water content. The synergistic covalent crosslinking by TG and hydrogen/ionic interactions from citric acid construct a rigid, compact polymer framework that hinders thermal chain decomposition, thereby significantly boosting the overall thermal stability of composite films.

Many previous studies on TG claim that crosslinking reaction reduces the bound water content inside biopolymer films. However, the higher-temperature-shifted and intensified DTG water-loss peak of dual-crosslinked SPI–CS film clearly demonstrates that dense dual-crosslink networks lock more bound water molecules inside the matrix. This revised interpretation of thermal mass-loss behavior is a unique observation of this study.

It should be noted that the thermal analysis in this work was based on TG/DTG; a quantitative DSC characterization of the glass transition temperature and denaturation enthalpy was beyond the scope of this study, which focuses on the preparation, structural characterization and application performance of composite films.

### 3.7. Mechanical and Antioxidant Properties of Antioxidant-Loaded SPI–CS Film

**Figure 12:**
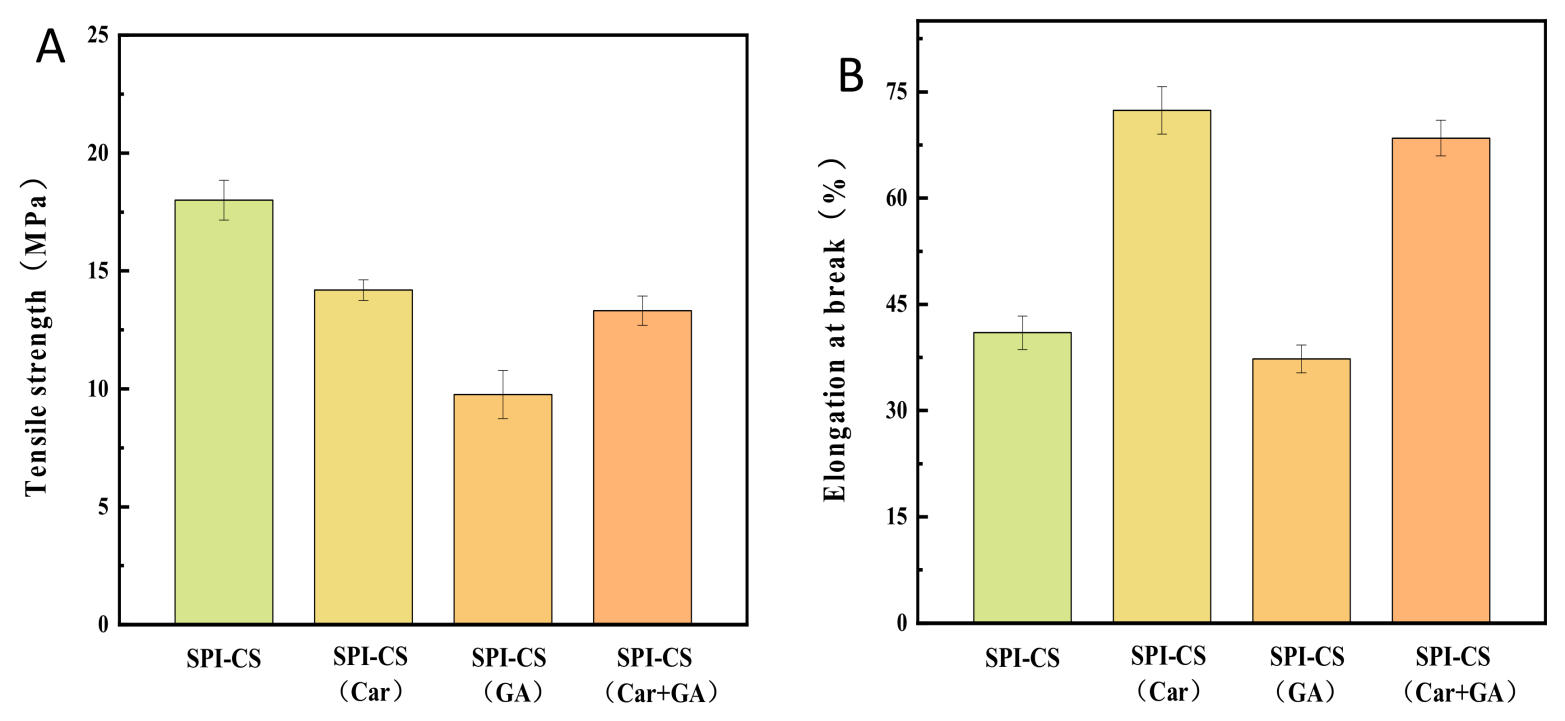
Mechanical properties of different SPI–CS films. (**A**) Tensile strength; (**B**) Elongation at break.

Figure 12 presents the mechanical properties of the SPI–CS films loaded with antioxidants. Figure 12A shows their tensile strength (TS): carvacrol group (14.19 MPa), GA group (9.76 MPa), and composite group (13.32 MPa). All the experimental groups have lower mechanical resistance than the control, as antioxidants weaken protein hydrogen bonds [50], damage the SPI–CS crosslinked network, and reduce rigidity/TS—a change mitigated by the two antioxidants’ synergistic effect. Figure 12B reveals distinct impacts on elongation at break (EAB): carvacrol acts as a plasticizer, increasing protein-chain mobility and EAB to 72.37%; GA occupies more voids, reducing component crosslinking and slightly lowering EAB [51]. Overall, carvacrol alleviates GA’s negative effects on mechanical strength and flexibility, and the antioxidant composite system enhances the comprehensive performance of SPI–CS films.

**Figure 13:**
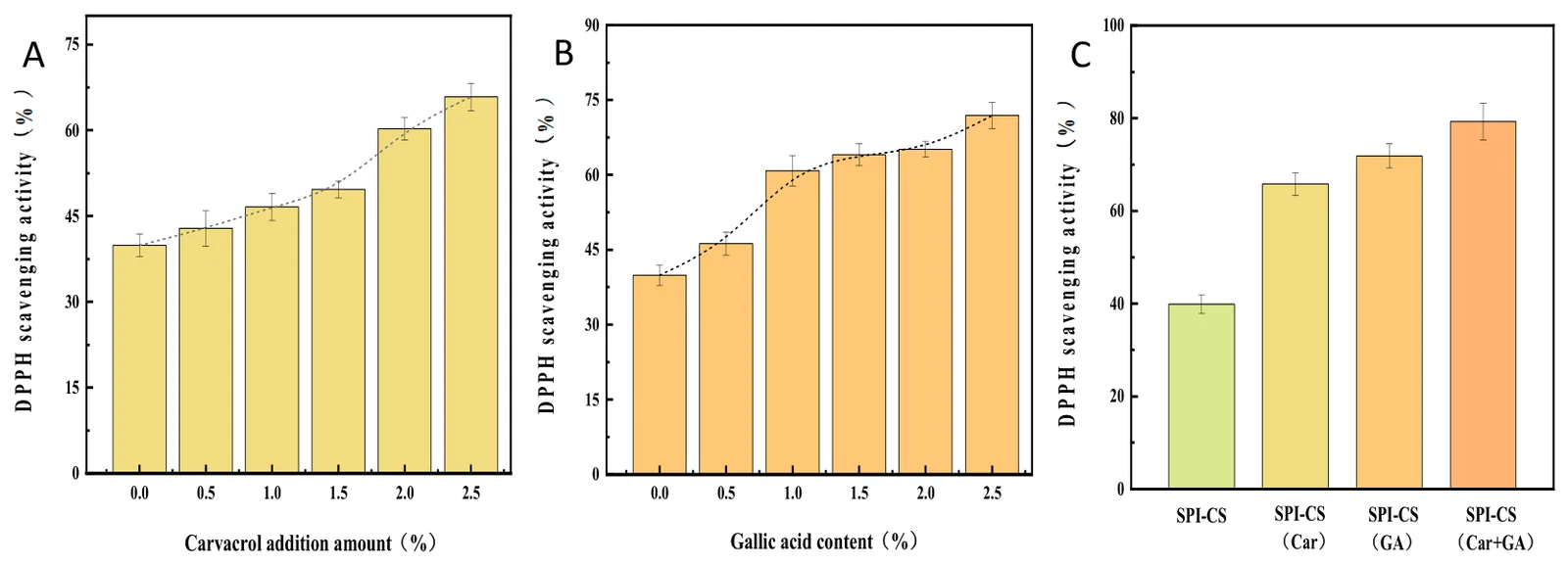
Key Factors Affecting the Antioxidant Properties of Composite Films. (**A**) Effect of Different Carvacrol Dosages on the Antioxidant Properties of Composite Films; (**B**) Effect of Different Gallic Acid Dosages on the Antioxidant Properties of Composite Films; (**C**) Antioxidant Properties of Different SPI–CS Films.

Car and gallic acid, both natural antioxidants, significantly enhanced the DPPH radical-scavenging capacity of SPI–CS composite films [52]. As shown in Figure 13A, increasing the carvacrol content from 0% to 2.5% raised the DPPH radical-scavenging rate from 39.88% to 65.83%; the abundant phenolic hydroxyl groups of carvacrol can capture free radicals via hydrogen donation. As displayed in Figure 13B, gallic acid exhibited stronger antioxidant activity: the scavenging rate rose rapidly within the 0–1.0% addition range and reached 70.89% at a 2.5% dosage, attributed to its three ortho-phenolic hydroxyl groups with high hydrogen supply efficiency [53,54].

Notably, the SPI–CS film co-loaded with 2.5% carvacrol and 2.5% gallic acid only achieved a DPPH radical-scavenging rate of 79.33% (Figure 13C). The theoretical additive scavenging value, calculated on a net-effect basis after subtracting the blank-film baseline (E_theory = E_Car + E_GA − E_blank = 65.83% + 70.89% − 39.88% = 96.84%), is markedly higher than the measured 79.33%; a Bliss independence model gives a similar estimate (1 − (1 − 0.6583)(1 − 0.7089) = 90.05%). The measured value being lower than both theoretical estimates confirms the absence of antioxidant synergism (a non-additive response). Most relevant published literature reports obvious synergistic antioxidant effects after mixing carvacrol and gallic acid, which is inconsistent with our experimental observation [27,29,48]. We speculate that competitive hydrogen-donating reactions occur between the phenolic hydroxyl groups of carvacrol and gallic acid, reducing the overall radical neutralization efficiency. It should be noted that the DPPH assay was performed on the 24 h aqueous extract and therefore reflects the Car/GA fraction released into water rather than the total film antioxidant content; the 39.88% baseline arises from intrinsic reducing groups of the SPI/CS matrix. The partial/slow aqueous release of hydrophobic carvacrol relative to water-soluble gallic acid may also contribute to the sub-additive measured value. Release kinetics were not quantified. This research provides new supplementary experimental data for the compound application of plant polyphenols in bio-based packaging materials.

### 3.8. Application Analysis of Soy Protein–Chitosan Antioxidant Film

**Figure 14:**
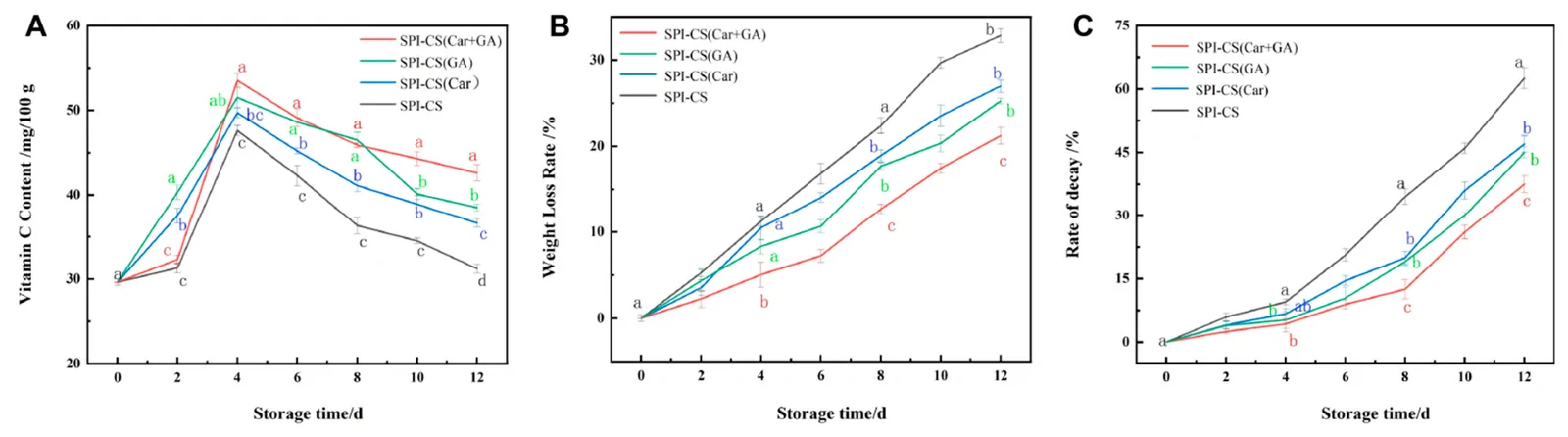
Variations in (**A**) vitamin C content, (**B**) weight loss rate and (**C**) rot index of cherry tomatoes under different film packaging during storage. Different lowercase letters indicate significant differences (*p* < 0.05, one-way ANOVA followed by Tukey HSD).

The fresh-keeping performance of SPI–CS composite films was assessed via three core fruit quality indices over a 12-day storage period: the residual vitamin C content, weight loss rate, and fruit rot index (Figure 14). Four packaging groups were set for comparison: pure SPI–CS blank film, carvacrol single-loaded film, gallic acid single-loaded film, and dual carvacrol/gallic acid co-loaded composite film with a DPPH radical-scavenging rate of 79.33%.

As illustrated in Figure 14A, the Vc content of cherry tomatoes from all groups rose in the early ripening stage and declined afterwards, with peak values ranging from 47.6 to 53.5 mg per 100 g fresh weight. On Day 12 of storage, fruits wrapped in the dual-antioxidant film retained 42.6 mg/100 g Vc, which was remarkably higher than the 31.2 mg/100 g measured for the blank group. This result confirms that the dual-polyphenol system effectively delays oxidative decomposition of endogenous ascorbic acid in cherry tomatoes [23,55,56].

Figure 14B depicts the dynamic weight loss trend of samples during storage. The incorporation of natural polyphenols significantly retarded fruit water transpiration. After 12 days of storage, the dual-antioxidant film group exhibited the lowest weight loss rate of 21.22%, whereas the blank SPI–CS film group reached 32.82%.

For fruit rot performance (Figure 14C), the rot index of the dual-antioxidant treatment only reached 37.5% by Day 12, while the blank group suffered a rot ratio as high as 62.5%. The reduced rot index is consistent with the individually documented antibacterial activities of carvacrol and gallic acid, which can disrupt microbial membranes and suppress proliferation; however, as direct microbial enumeration was not performed, a specific synergistic bacteriostatic action cannot be inferred from the rot-index data alone.

**Figure 15:**
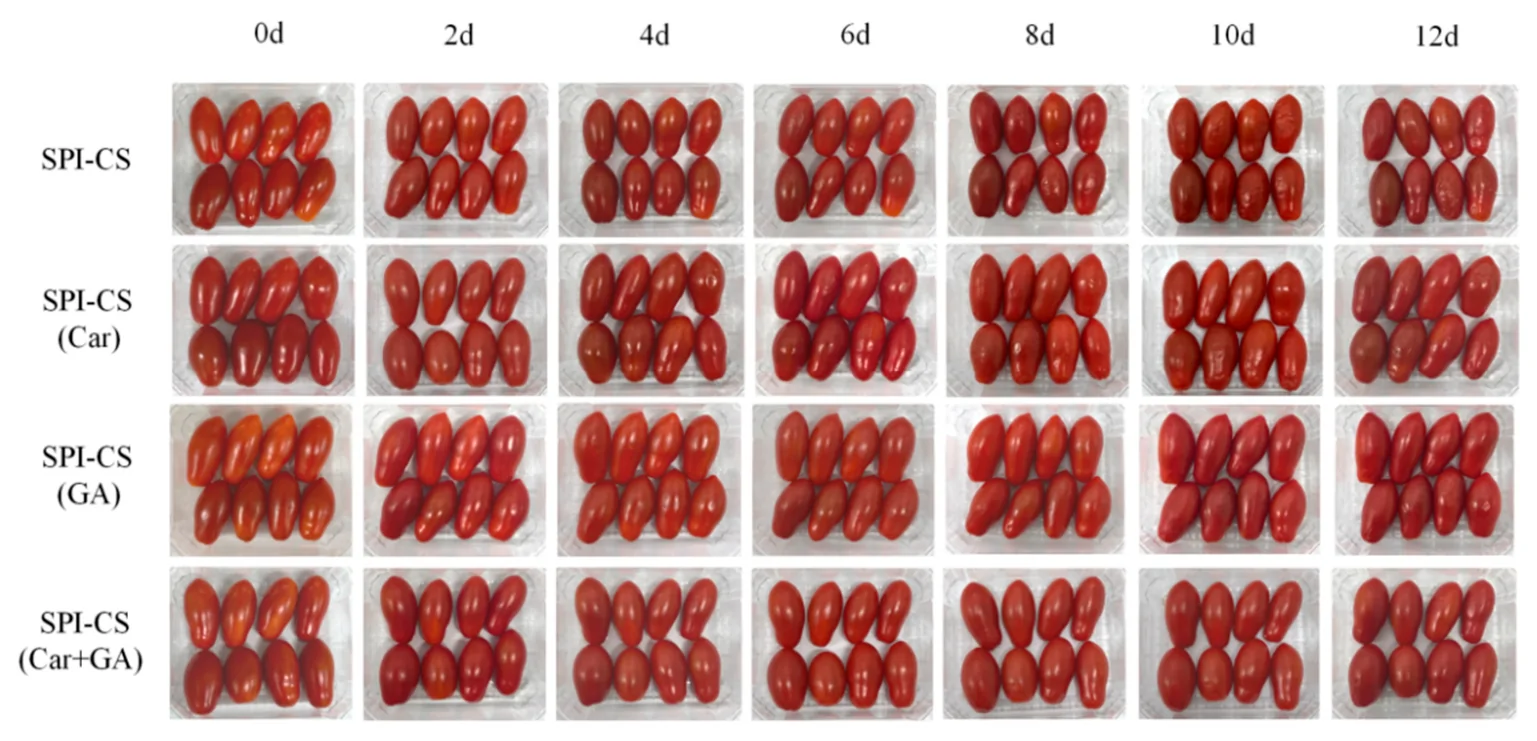
Visual photographs of cherry tomatoes at different storage time points.

Figure 15 combines macroscopic fruit photographs and quantitative sensory scores recorded at 2-day intervals (Day 0, 2, 4, 6, 8, 10, 12). A standardized 9-point hedonic scale was adopted by ten trained assessors, and all samples were randomly coded to avoid subjective evaluation bias. Fruits packed with single-antioxidant films consistently achieved higher sensory scores than blank samples, and the dual-polyphenol composite film delivered the best overall preservation effect: distinct epidermal shriveling was only observed starting from Day 8, and nearly half of the fruits still retained complete marketable appearance and acceptable sensory attributes at the end of storage.

In summary, SPI–CS films co-incorporated with carvacrol and gallic acid (79.33% DPPH radical-scavenging activity) exhibit excellent comprehensive fresh-keeping capacity. Despite the absence of antioxidant synergism between the two polyphenols, the presence of both polyphenols, combining radical-scavenging activity with individually documented antibacterial effects, alleviates fruit oxidative deterioration and microbial spoilage. The dual-functional film stabilizes endogenous Vc levels, reduces water loss, slows tissue decay, and maintains the original color, texture and shape of cherry tomatoes, thus greatly extending their shelf life.

## 4. Discussion

The structural and spectroscopic results indicate that the ultrasonic-assisted inorganic ternary DES acts primarily by disrupting the non-covalent and covalent interactions that maintain the compact SPI architecture rather than by chemically modifying the polypeptide. Attenuation of the O–H/N–H and disulfide bands, together with the loss of ordered domains, is consistent with the hydrogen-bond competition and disulfide-cleavage effects reported for other physical and solvent-based protein treatments; in those studies, as here, the amide I–III bands were retained, confirming that unfolding and deagglomeration, rather than backbone degradation, dominate. With a comparable starting crystallinity (~20%), the larger relative decrease observed here (25.4%) indicates that the metal-ion/phosphoric-acid system unfolds aggregates more effectively than the choline-based and single-denaturant systems reported previously, while avoiding organic hydrogen-bond donors.

A central finding is that, under the mild 65 °C film-forming condition, citric acid does not establish ester crosslinks with the SPI/CS hydroxyl groups. This agrees with the thermal requirements of citrate esterification: ester and amide bond formation between citric acid and soybean flour has been reported only after heating at about 140 °C with a catalyst, as verified by a new ester carbonyl band near 1711 cm^−1^ [57]. Consistent with this, no new band appeared anywhere in the ester carbonyl region (1700–1750 cm^−1^; a citrate ester C=O would be expected near 1730–1750 cm^−1^) in the present spectra, indicating that citric acid contributes through multi-carboxyl ionic and hydrogen-bond bridges, whereas the covalent network is supplied mainly by TG-catalyzed isopeptide bonds among SPI chains. This clarifies a mechanism that is often misassigned as citrate ester crosslinking in mild-temperature protein films.

Although carvacrol and gallic acid are individually effective radical scavengers, their combination showed no synergism: the measured DPPH scavenging (79.33%) was well below the theoretical additive values (96.84% on a net-effect basis; 90.05% by the Bliss independence model). Such antagonism is not unique; pairwise phenolic acids have likewise given activities below their arithmetic sum in the DPPH assay, and the effect has been linked to differing hydrogen-transfer ability and to competition for the radical [58]. The two polyphenols most likely compete as hydrogen donors and for access to the DPPH radical, lowering their combined efficiency. This qualifies the common assumption that mixing phenolic antioxidants is automatically beneficial and adds a specific counterexample for bio-based packaging.

Despite the lack of in vitro antioxidant synergism, the co-loaded film gave the best preservation of cherry tomatoes, retaining more vitamin C and showing the lowest weight loss and rot index with acceptable sensory quality. Practical performance is therefore attributed to the combined presence of the two polyphenols—each with documented antibacterial activity—together with the improved water resistance and structural integrity of the dual-crosslinked matrix, rather than to additive radical scavenging alone. The route is water-based, formaldehyde-free, conducted under mild ambient-pressure conditions, and the matrix is biodegradable.

Several limitations should be acknowledged. First, residual DES components (Zn^2+^, Cl^−^ and phosphate) and their migration into food simulants were not quantified; despite repeated water washing, the films therefore cannot be designated as edible or food-contact safe, and such a safety assessment is a prerequisite for food-related use. Second, several dedicated characterizations were not performed—water-vapor and O_2_/CO_2_ transmission rates (barrier performance supported only indirectly by solubility, contact angle and packaged-fruit data), film DSC (thermal behavior assessed via TG/DTG, FTIR and mechanics), DSC confirmation of the solvent eutectic point (the DES classification resting on formulation rather than solid–liquid equilibrium), and direct microbial enumeration (antibacterial activity inferred only from the rot index). Third, the dry-mass recovery yield of the pretreated precipitate was not quantified, so the relationship between the supernatant soluble-protein content and the actual precipitate yield remains to be established. Fourth, an unwrapped control was absent, so the absolute barrier contribution relative to no packaging was not quantified (the polyphenol-free blank film nevertheless isolates the polyphenol effect). Finally, quantitative verification of the crosslink network, together with processing stability and cost at larger scale, remains to be addressed.

## 5. Conclusions

In this study, a multifunctional SPI–CS composite film was developed by combining ultrasonic-assisted ternary DES pretreatment with citric acid/transglutaminase dual crosslinking. The main findings are as follows.

(1) The low-cost inorganic ZnCl_2_-H_3_PO_4_-H_2_O DES coupled with ultrasound effectively deagglomerated SPI and unfolded its ordered domains (relative ordered-domain content decreasing from 20.57% to 15.34%) without damaging the protein primary structure.

(2) Under the mild 65 °C forming condition, no ester bond formation between citric acid and the SPI/CS hydroxyl groups was detectable by FTIR; the dual-crosslink network was established mainly through TG-catalyzed covalent isopeptide bonds among SPI chains (with possible SPI–chitosan links), together with citric-acid-mediated ionic and hydrogen-bond interactions.

(3) Co-incorporation of carvacrol and gallic acid gave a DPPH scavenging rate of 79.33%, which is lower than the theoretical additive value of 96.84%, indicating no antioxidant synergism, while the dual-antioxidant film still achieved effective long-term preservation of cherry tomatoes.

The films are produced in a water-based and biodegradable system under mild conditions and show promise as sustainable bio-based packaging candidates, subject to confirmation of residual solvent levels and migration compliance before food-contact use.

## Figures and Tables

**Table 1:** Full names and abbreviations of all film samples.

Abbreviation	Full Name
CS film	Pure chitosan film
SPI film	Pure native soy protein isolate film
DES–SPI film	Deep eutectic solvent-pretreated soy protein isolate film
Uncrosslinked SPI–CS film	Uncrosslinked soy protein isolate–chitosan composite film
Crosslinked SPI–CS film	Soy protein isolate–chitosan composite film dual-crosslinked by citric acid and TG
SPI–CS-Car film	Crosslinked SPI–CS film loaded with carvacrol
SPI–CS-GA film	Crosslinked SPI–CS film loaded with gallic acid
SPI–CS-Car+GA film	Crosslinked SPI–CS film loaded with both carvacrol and gallic acid

**Table 2:** Coding of factor and level of Box–Behnken experimental design.

Level	Factor A: Citric Acid Mass Ratio	Factor B: TG Addition (U∙g^−1^)	Factor C: Film-Formation Temperature (°C)
−1.0	0.5	2	45
0	1.0	3	55
1.0	1.5	4	65

**Table 3:** Response Surface Experimental Design and Results.

Code	Sample ID	Mass Ratio	TG Dosage (U∙g^−1^)	Film-Forming Temperature(°C)	Tensile Strength (MPa)	Elongation at Break (%)
4	1	1.5	4	55	10.60 ± 0.7	57.43 ± 3.2
6	2	1.5	3	45	13.45 ± 0.8	50.36 ± 2.9
1	3	0.5	2	55	10.53 ± 0.6	45.75 ± 2.6
13	4	1	3	55	15.18 ± 1.0	45.26 ± 2.8
2	5	1.5	2	55	12.89 ± 0.8	61.50 ± 3.4
10	6	1	4	45	8.29 ± 0.6	30.55 ± 2.1
7	7	0.5	3	65	17.85 ± 1.1	37.58 ± 2.4
11	8	1	2	65	18.94 ± 1.1	49.07 ± 2.8
16	9	1	3	55	13.02 ± 0.9	36.78 ± 2.5
3	10	0.5	4	55	9.48 ± 0.6	40.42 ± 2.5
9	11	1	2	45	13.52 ± 0.8	33.43 ± 2.2
15	12	1	3	55	12.59 ± 0.8	46.61 ± 2.8
12	13	1	4	65	16.17 ± 1.0	38.41 ± 2.4
14	14	1	3	55	11.52 ± 0.7	43.84 ± 2.7
5	15	0.5	3	45	6.93 ± 0.5	30.51 ± 2.1
17	16	1	3	55	14.45 ± 0.9	38.46 ± 2.5
8	17	1.5	3	65	15.90 ± 0.9	59.18 ± 3.3

**Table 4:** Chromaticity comparison of different film materials.

Sample	Chrominance				Sample State
	L*	a*	b*	△E*ab	
CS	88.54	0.65	−2.75	3.78	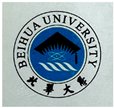
DES–SPI	81.21	−0.64	10.788	19.13	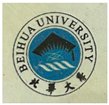
SPI–CS Plain Membrane	84.02	−0.23	2.96	11.12	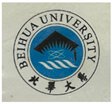
SPI–CS Crosslinked Membrane	85.11	−0.01	1.56	9.32	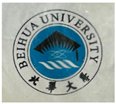

## Data Availability

The data presented in this study are available on request from the corresponding author. The data are not publicly available due to ongoing research based on these data.

## Supplementary Materials

The following supporting information can be downloaded at: https://www.mdpi.com/article/10.3390/polym18212743/s1.
