# Supplementary material for: Preparation and Properties of Deep Eutectic Solvent-Modified Soy Protein and Soy Protein–Chitosan Composite Films
Source: Polymers (Basel). 2026 Nov 4;18(21):2743. doi: 10.3390/polym18212743 (PMC13765603; doi:10.3390/polym18212743)
Supplement: Supplementary file 1 [file polymers-18-02743-s001.zip › polymers-4571315-supplementary.pdf]

1.Response Surface Regression Model for Tensile Strength

Analysis of variance (ANOVA) of the mathematical regression model was carried out via Design Expert software, and the results are summarized in Table S1. The P-value is used to judge the statistical significance of each term on the response value: $P > 0.05$ indicates a non-significant effect, $P < 0.05$ denotes a significant effect, and $P < 0.01$ corresponds to an extremely significant effect. The overall model P-value was calculated as 0.0024 ($P < 0.01$), demonstrating that the regression model exerted an extremely significant influence on the tensile strength of composite films. The lack-of-fit term exhibited a P-value of 0.7345 ($P > 0.05$), which was statistically non-significant, verifying the satisfactory fitting performance and validity of the established model for predicting tensile strength under various preparation conditions.

As listed in Table S1, the P-value of transglutaminase (TG) dosage was less than 0.05, and the P-value of film-forming temperature was lower than 0.01, revealing that these two factors imposed significant and extremely significant impacts on tensile strength, respectively. Meanwhile, the interaction between citric acid mass ratio and film-forming temperature presented a significant effect ($P < 0.05$). Comprehensive comparison of significance levels ranks the influencing factors on tensile strength in the following order: film-forming temperature > citric acid mass ratio > TG dosage.

In general, a coefficient of determination R^2 greater than 0.8 represents favorable fitting quality of the response surface regression equation, and a value closer to 1 indicates stronger correlation between experimental data and predicted values. The R^2 of the present model reached 0.9327, close to unity, which confirms excellent data fitting performance. Adeq Precision=11.5683, far exceeding the critical threshold of 4, manifesting a high signal-to-noise ratio and reliable model practicability. The adjusted coefficient of determination Adj R^2=0.8462, reflecting strong correlation among all investigated variables, while the predicted coefficient of determination Pred R^2=0.6520, demonstrating satisfactory predictive capacity of the established regression model.

Table S1. Estimation regression model of the relationship between response variables and influencing factors

Source	Sum of Squares	df	Mean Square	F-value	P-value	Evaluation
Model	158.78	9	17.64	10.78	0.0024	significant
A-Citric acid mass ratio	8.10	1	8.10	4.95	0.0615	
B-TG enzyme dosage	16.07	1	16.07	9.82	0.0165	
C-Film-forming temperature	88.91	1	88.91	54.32	0.0002	
AB	0.3844	1	0.3844	0.2348	0.6428	
AC	17.94	1	17.94	10.96	0.0129	
BC	1.51	1	1.51	0.9243	0.3684	

A2	10.61	1	10.61	6.48	0.0383	
B2	3.33	1	3.33	2.04	0.1966	
C2	13.16	1	13.16	8.04	0.0252	
Residual	11.46	7	1.64			
Lack of Fit	2.86	3	0.9545	0.4443	0.7345	not significant
Pure Error	8.59	4	2.15			
Cor Total	170.24	16				

$R2=0.9327$，Adj $R2=0.8462$，Pred $R2=0.6520$，Adeq Precision=11.5070

Multiple quadratic regression analysis was performed on the tensile strength response data listed in Table S1, and the fitted regression equation is presented as follows:

Tensile Strength

$=13.35+1.01A-1.42B+3.33C-0.3100AB-2.12AC+0.6150BC-1.59A^2-0.8898B^2+1.77C^2$

The normal probability plot of residuals, residual vs. predicted value plot, and predicted value vs. actual experimental value plot are displayed in Figure S1, Figure S2 and Figure S3, respectively. All data points fall closely along a straight line in the normal probability plot of residuals; the scatter points in the plot of predicted versus actual values are distributed near a straight line; and no obvious regular distribution trend is observed for data points in the residual-predicted plot. These results demonstrate that the residuals follow a normal distribution, the experimental data are reliable, and the established quadratic regression model possesses excellent adaptability and fitting performance.

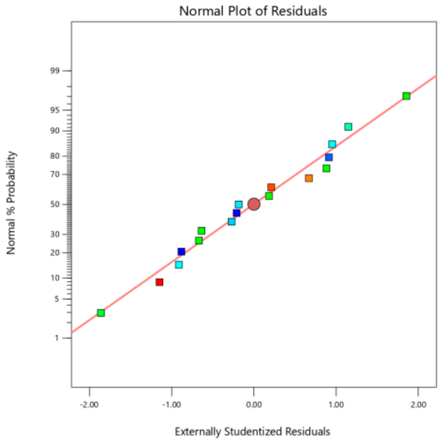

Figure.S1 Normal probability distribution plot

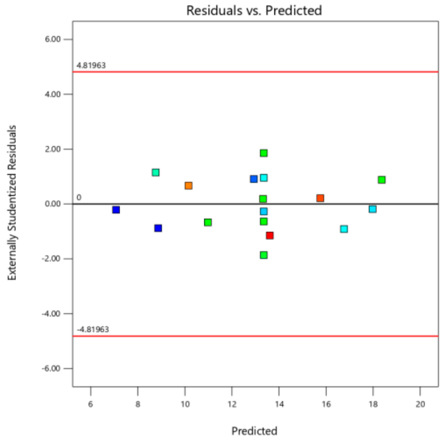

Figure.S2 Distribution diagram of residual and predicted values

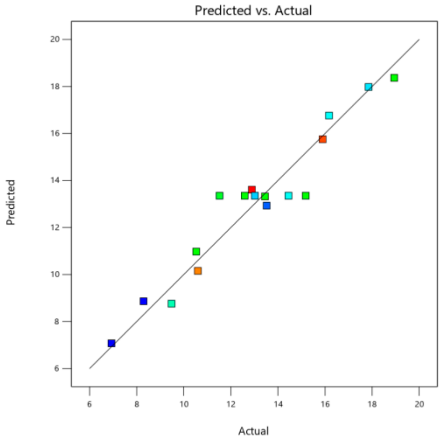

Figure.S3 Distribution chart of predicted value and actual value

2.Response Surface Regression Model for Elongation at Break

Table S2 presents the ANOVA results of the quadratic regression model fitted for elongation at break. The P-value is adopted to evaluate the statistical significance of each factor's influence on the response variable. The overall model P-value was 0.0022, which was less than 0.01, indicating that the regression model exerted an extremely significant effect on the elongation at break of composite films. The P-value of the lack-of-fit term was 0.8020 ($P > 0.05$), demonstrating non-significant lack of fit, which verifies the favorable fitting quality and valid predictive capability of the established model for elongation at break under various preparation conditions. As shown in Table S2, both the mass ratio of citric acid and film-forming temperature had P-values below 0.01, revealing that these two factors imposed extremely significant impacts on the elongation at break response. The factors affecting

elongation at break are ranked in the order: citric acid mass ratio > film-forming temperature > TGase dosage.

In general, a coefficient of determination (\(R^2\)) greater than 0.8 indicates satisfactory fitting performance of the response surface regression equation, and a value closer to 1 reflects stronger correlation between experimental data and model predictions. In this experiment, the R^2 reached 0.9348, close to unity, which proves excellent data fitting of the established model. The Adeq Precision=11.5683, far exceeding the critical threshold of 4, manifesting a high signal-to-noise ratio and reliable practicability of the regression model. The adjusted coefficient of determination Adj R^2=0.8510, which implies strong correlation among all investigated variables, while the predicted coefficient of determination Pred R^2=0.7090, demonstrating the outstanding predictive performance of the model.

Table.S2 Estimation regression model of the relationship between response variables and influencing factors

Source	Sum of Squares	df	Mean Square	F-value	P-value	Evaluation
Model	1343.23	9	149.25	11.15	0.0022	significant
A-Citric acid mass ratio	688.39	1	688.39	51.44	0.0002	
B-TG enzyme dosage	65.78	1	65.78	4.91	0.0622	
C-Film-forming temperature	193.95	1	193.95	14.49	0.0067	
AB	0.3969	1	0.3969	0.0297	0.8681	
AC	0.7656	1	0.7656	0.0572	0.8178	
BC	15.13	1	15.13	1.13	0.3229	
A2	257.07	1	257.07	19.21	0.0032	
B2	6.80	1	6.80	0.5084	0.4989	
C2	131.87	1	131.87	9.85	0.0164	
Residual	93.69	7	13.38			
Lack of Fit	18.82	3	6.27	0.3352	0.8020	not significant

Pure Error	74.86	4	18.72
Cor Total	1436.92	16	

$R2=0.9348$，Adj $R2=0.8510$，Pred $R2=0.7090$，Adeq Precision=11.5683

Multiple quadratic regression analysis was performed for the response of elongation at break based on Table S2, and the fitted regression equation is expressed as follows:

Elongation at Break

$$=42.19+9.28A-2.87B+4.92C+0.3150AB+0.4375AC-1.75BC+7.81A^2+1.27B^2-5.60C^2$$

The normal probability plot of residuals, residual versus predicted value plot, and predicted value versus actual experimental value plot are presented in Figure S4, Figure S5 and Figure S6, respectively. All data points fall closely along a straight line in the normal probability plot of residuals; scatter points are distributed adjacent to a straight line in the plot of predicted values against actual measurements; and no obvious regular distribution trend is observed for data points in the residual-predicted plot. These results demonstrate that the residuals follow a normal distribution, the experimental dataset is credible, and the established regression model exhibits excellent adaptability and fitting performance.

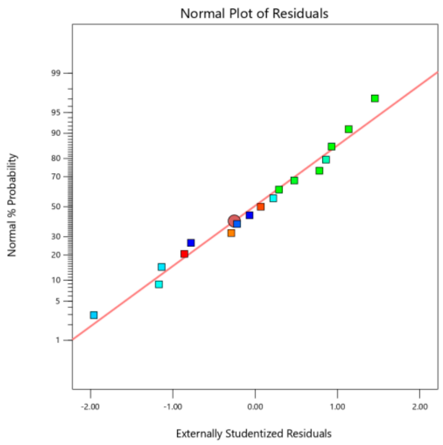

Figure.S4 Normal probability distribution plot

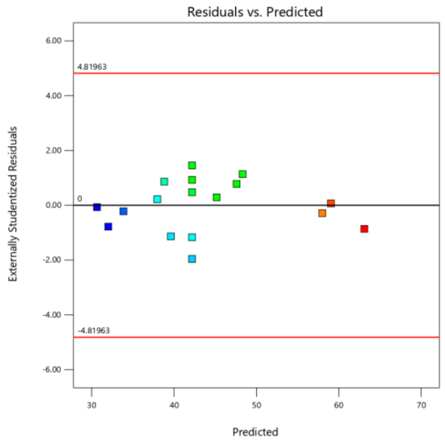

Figure.S5 Distribution diagram of residual and predicted values

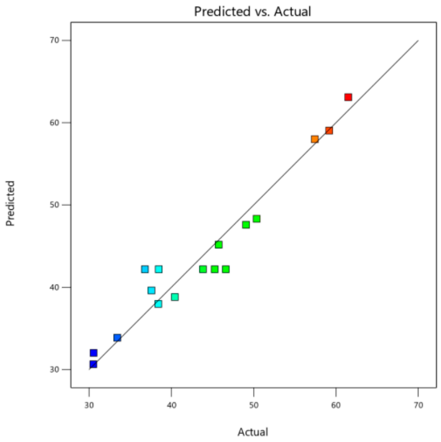

Figure.S6 Distribution chart of predicted value and actual value
